# A 124-plex Microhaplotype Panel Based on Next-generation Sequencing Developed for Forensic Applications

**DOI:** 10.1038/s41598-020-58980-x

**Published:** 2020-02-06

**Authors:** Jing-Bo Pang, Min Rao, Qing-Feng Chen, An-Quan Ji, Chi Zhang, Ke-Lai Kang, Hao Wu, Jian Ye, Sheng-Jie Nie, Le Wang

**Affiliations:** 1 National Engineering Laboratory for Forensic Science, Institute of Forensic Science, Ministry of Public Security, Beijing, 100038 PR China; 2 Key Laboratory of Forensic Genetics of Ministry of Public Security, Institute of Forensic Science, Ministry of Public Security, Beijing, 100038 PR China; 3 School of Forensic Medicine, Kunming Medical University, Kunming, 650500 PR China

**Keywords:** Molecular medicine, Next-generation sequencing

## Abstract

Microhaplotypes are an emerging type of forensic genetic marker that are expected to support multiple forensic applications. Here, we developed a 124-plex panel for microhaplotype genotyping based on next-generation sequencing (NGS). The panel yielded intralocus and interlocus balanced sequencing data with a high percentage of effective reads. A full genotype was determined with as little as 0.1 ng of input DNA. Parallel mixture experiments and in-depth comparative analyses were performed with capillary-electrophoresis-based short tandem repeat (STR) and NGS-based microhaplotype genotyping, and demonstrated that microhaplotypes are far superior to STRs for mixture deconvolution. DNA from Han Chinese individuals (n = 256) was sequenced with the 124-plex panel. In total, 514 alleles were observed, and the forensic genetic parameters were calculated. A comparison of the forensic parameters for the 20 microhaplotypes with the top A_e_ values in the 124-plex panel and 20 commonly used forensic STRs showed that these microhaplotypes were as effective as STRs in identifying individuals. A linkage disequilibrium analysis showed that 106 of the 124 microhaplotypes were independently hereditary, and the combined match probability for these 106 microhaplotypes was 5.23 × 10^−66^. We conclude that this 124-plex microhaplotype panel is a powerful tool for forensic applications.

## Introduction

The microhaplotype is a powerful new type of forensic genetic marker^1,2^. It is the combination of two or more closely linked single-nucleotide polymorphisms (SNPs) within DNA segments of 200 base pairs (bp), and offers multiple forensic applications^3–7^. Short tandem repeat (STR) genotyping is currently the dominant technology in forensic DNA laboratories. Although it works well with single-sourced DNA samples, great challenges are encountered with DNA mixtures because stutters in the major donor DNA can be indistinguishable from alleles in the minor donor DNA^3,8^. Stutters are unavoidable during the replication of repetitive DNA, and they severely interfere with mixture deconvolution. SNPs are not repetitive sequences, but are typically biallelic, which restricts their utility in the analysis of mixtures. Microhaplotypes have the advantages of both STRs and SNPs because they are multiallelic and do not produce stutters during amplification. Therefore, microhaplotypes are perfect genetic markers for mixture deconvolution.

Although capillary electrophoresis (CE)-based genetic analyzers are widely used in forensic DNA laboratories, these machines are unsuitable for microhaplotype genotyping^8^. Several methods have been used for microhaplotype detection. TaqMan assays have been used to type each SNP that constitutes a microhaplotype^8^, followed by a PHASE software analysis to determine the *cis*/*trans* relationships between individual SNP alleles. Single-strand conformational polymorphisms^9^ and high-resolution melting curves^4^ have also been used for microhaplotype genotyping. These methods are simple and inexpensive, but they can pose problems when multiplexing different loci or dealing with mixed samples. MinION, a nanopore sequencing machine, has also been used for microhaplotype sequencing^10^, but the accuracy of sequencing for forensic applications must be improved.

Next-generation sequencing (NGS) is well accepted by the forensic community. Both the Illumina and Ion Torrent sequencers are high throughput, with appropriate read lengths for microhaplotypes^11,12^, and NGS can directly determine the phase between SNP alleles. Based on these characteristics, NGS is considered the optimal strategy for microhaplotype genotyping, and the development of NGS has made microhaplotypes a powerful new type of genetic marker for forensic analyses^2^. Zhu *et al*.^13^, Qu *et al*.^14^, Turchi *et al*.^12^, and Kidd *et al*.^15^ have studied microhaplotypes for forensic applications on the Miseq, HiSeq, Ion Personal Genome Machine (PGM), and Ion S5™ platforms, respectively. Attempts to develop NGS-based microhaplotype panels and microhaplotype population data have also been reported. In 2017, 89 microhaplotypes were sequenced with two primer pools in 73 Italian samples^12^, and this panel was later optimized to 87 loci by the same research group^16^. Another research team constructed a 74-plex microhaplotype assay and sequenced 278 samples from three different populations^15^. In the present study, we developed and evaluated a multiplex amplification system containing 124 microhaplotype loci. Parallel mixture experiments were performed with CE-based STR and NGS-based microhaplotype genotyping methods to compare their capacities for forensic mixture deconvolution. Microhaplotype allelic diversity and forensic estimations were determined for a Han Chinese population.

## Results

### The 124-plex microhaplotype panel

A total of 124 microhaplotype loci were multiplexed in a single primer pool. The number of SNPs contained at each locus ranged from 2 to 5, and 52 loci contained ≥ 3 SNPs (Supplementary Table S1). The molecular extent of the loci ranged from 13 to 210 nt, with an average of 108 nt. The primer sequences, primer concentrations, and amplicon sizes of the 124-plex panel are summarized in Table 1. The amplicons ranged from 63 to 298 bp, with an average size of 212 bp (Supplementary Fig. S1).

**Table 1:** The 124 microhaplotype loci and the related parameter information of primers.

Locus name	Primers for PCR amplification	C	AS	Locus name	Primers for PCR amplification	C	AS
mh01KK-002	TCTGGATAAGGGAGGAAGAAACT	0.20	135	mh11KK-037	TTTCCATCTCACCAGGCATCA	0.08	222
	GCCTTCTAGTTCTGAAGCCAATAT				CCTGGGATAACAGGAAAGAAATC		
mh01KK-070	CCCACTCCAGCATCACTCAC	0.04	152	mh11KK-038	CCCAGGGTTGTTGCTTCCA	0.08	269
	TTCTACCTGAAGAGCAAGTCCC				CTCTAAAACCCGACGCTGC		
mh01KK-072	CCCTTTTCCGAATTTTCCTG	0.08	115	mh11KK-039	TGTTCCTGCCAAACCATTCA	0.04	197
	GTATTCCCCTACTTTGTCTTCTGG				GACCTCGTTGTCACTGATGATACTA		
mh01KK-106	ATCCAGTCCCGCTGCCTG	0.04	244	mh11KK-040	TGAACTTCCTGCACAGCATTAA	0.04	126
	GATGTCAGATTTTCTTAGGACCGA				AAGTGAAAGGGAGCGGAGG		
mh01KK-117	GTCTCCCCACAAAGCATTGC	0.04	243	mh11KK-041	GCAATCTTGGGGTGGTCTTT	0.04	91
	GGTCACATCACCATCTCCGTC				CCGACCCGTCCCACCA		
mh01KK-205	TAGAAGAAAGCACTAATGGGGTAAT	0.04	248	mh11KK-089	ACCTGCTCTGCTCACCTAACTCA	0.04	115
	CAATTCGCAACAGTGAAAGCAT				GGATGCCTCCTGTGCCTGTA		
mh01KK-210	TCCAGAGTGGTTTGCAGGC	0.04	278	mh11KK-090	GTTGAGTCTGGGGAGGTTGC	0.02	150
	AAGTAATTGGCTCCAGGTGACA				CTCCGTTCTCCACAGTGCTG		
mh01KK-211	AGATCAAGTCGGCCACGATG	0.04	243	mh11KK-091	CCCACCAAAGGAGCTGTACC	0.20	190
	CACCTCCTCCATAATCCACAAGT				GGAGAAGACTGGCGAGCAGA		
mh02KK-003	TGTGCAATGAAGAGCTAACTTGTG	0.04	178	mh11KK-180	GACCTGCCTGCTTTTCCTGA	0.08	288
	GCTGGGCTGGCTAGACCCT				TTGCACCCTCGCTTCCC		
mh02KK-005	GCTGGGCCCTAACAGTCTCA	0.08	259	mh11KK-187	CTGACTGTCAGCACTCCAGTATCA	0.04	250
	CAACAGCCATTGACTTTTCCC				TGGGTCTCGCCGCAAG		
mh02KK-073	TGGAAAATGGTTCTGAATCGG	0.04	127	mh11KK-191	GGGAAACAAAGGTATGTAAAGGC	0.04	296
	CACTTTATGGATTAACTCAACCTGG				CAGCAGTTCAGGCAAAGAGC		
mh02KK-102	ATCCTTAGTTGGGTAACCCTGTC	0.12	214	mh12KK-043	TCCTTAGGCAATGAGAAAACACTG	0.12	243
	AAATGCTCCTAGGTGAGTCTAATGT				GCAACCAAAAGAAGCCTCAGTC		
mh02KK-134	TTTGTGGCACTGGAGAACTG	0.04	198	mh12KK-045	GGTTATACCCTAAAACTAAAGTCTCGG	0.04	298
	CAATGTCCTTGAGGCTCGTAG				ATGTGCCTGCTCGTCTATCAA		
mh02KK-136	ATCCCCACTCCCCATGTTC	0.08	162	mh12KK-046	CAAATAGGAACACTGGTATAGGAGG	0.08	200
	CTCAGTATGTTTTGAGCACTTTCAG				TGGATTCAGGGGCATGGA		
mh02KK-201	TTTGAGTATGCTCTGTAGATGCTTC	0.12	169	mh12KK-092	TGGGGATGAACAGCTTGGA	0.20	182
	GAGTAACTGCTTCTCAAGTTGGAAT				TTGGTATGGCTTTGGCTAACTT		
mh02KK-202	GTGGGAGGGAACTTTCTGAGA	0.08	277	mh12KK-093	GCGTGATAGTGGCAATGATGG	0.04	236
	GTTGGGATTAGGGTTGGTATTG				CTTCTTACAGTTTCCTTGTTTCCGA		
mh02KK-213	CCCACCATTTGCCATGCT	0.04	236	mh12KK-202	TCCACCACCCACCTCTTCA	0.08	254
	CTCGGGTAGGGCTTTCTTTG				ACGTACAACCTGAGCCACTGAT		
mh03KK-006	TGACCGGACGCCATAGCC	0.04	132	mh13KK-047	ACAGTTACAACAAGAAGGAAATGGA	0.20	286
	GTCCTACATTACATGGTGTATAAAGCTCAG				GGGACGGGAAACAAATGATC		
mh03KK-007	TTTCAGTTTGTTCTTGGCAGC	0.12	94	mh13KK-213	GAGACAGCAAGGAGAACTTCAGTT	0.04	215
	TGCTGGAGATGTTATCAAGGCT				CTCAAATGGCGGGCTTCT		
mh03KK-008	CATGAACCTAGCAACAGACGAGC	0.20	272	mh13KK-217	TGCAAAATTTGGCTCAACAAGC	0.08	281
	GTGCAGAAAGATTCCAAAGGAGAAT				GGTGTATTGCCAAACAGAAAAGG		
mh03KK-009	GCCATTGCCGAAGACGAT	0.04	234	mh13KK-218	TAATAAAACTGGAATCATAAGCATAGC	0.08	209
	CAACCAAGCCCCAAAGAGTC				ACTAGAGTAATGCAGAACTCACATGTTA		
mh03KK-150	GTGCCATTTACTGACCACCTATTA	0.20	297	mh13KK-223	ACTAGAGTAATGCAGAACTCACATGTTA	0.08	280
	CCTGGGATCCACTGAAAGATT				TGACCAGCCTCTTTACATGGAGT		
mh04KK-010	TGAGCACAGAAGGAGCGATG	0.04	128	mh13KK-225	GAATTGGAGCTACAGCCACACT	0.08	203
	TGTGGGGTCACTTCAGGATAAT				CTGATGAAAAGGGAAGTGGAAA		
mh04KK-011	GTGTCTAATGGCCGCTGTAGTAA	0.04	142	mh13KK-226	AGTACAGTTTTCTCACCCCATAGG	0.08	191
	GCTCAGGAATTTTCATCTGCTTT				AATGGCTGTGGAAAGGGTAATA		
mh04KK-013	CATTGCAGTCATCTGAAATAAGCAC	0.04	250	mh14KK-048	GCCGTGGTGTCTGGAAAAC	0.12	231
	TTGGAAGCACCATACCACTCAG				GAGAAGCCAATGCAGGAGTCT		
mh04KK-015	TGGTCTGGTTTATTTTGGTTGG	0.04	226	mh14KK-068	TCTGTTCCATTGGCTCCTCTAC	0.04	158
	GGCAAAGGGGAATGACTGAG				CAGCTCACTTTTGCCCCTTT		
mh04KK-016	AGATTCAAGTTGAACTTTTAGACATCTG	0.12	196	mh14KK-101	CGGGATAAGGAATTAATCAAGGA	0.56	284
	TTTTCTTCCTAGGGCTACAATTACA				GCCATTAATATTTATTGTGATTACAACTG		
mh04KK-017	ATTGTACTGGTCGGATAATGAGC	0.04	290	mh15KK-066	CGGGACAAGGAATAGCCAGT	0.20	238
	ACTTCACTATACACTGGCTTTCTCC				CTTACCTGCCAACATATTCACCATA		
mh04KK-019	AACAATGATGCTACCTTCAGTGC	0.20	257	mh15KK-067	TTCTCCCCATTAAGCCATCCT	0.04	263
	ATTCTTATTTGGAAGATTACAACAGG				CCAGAAGAAGCAAAGACATCAAGA		
mh04KK-021	ACCACAGCGCCAAATGATG	0.04	282	mh15KK-095	CCCTAAACACCAGGATAGCAGTT	0.04	189
	GGAGGGGATCCTTTAGGACAGT				TTGAGGACGCTGCTGTTACTGT		
mh04KK-028	GCTGACTAATCTTGTGATGGTGAA	0.04	104	mh15KK-104	TTCCCACCTCACCTACATAATCT	0.08	240
	CGGCATCGTGGAAAGTGTT				GATGGAGCAGTAGTGATGAAGACA		
mh04KK-029	CTGATGGGTTTGGTAGAGTCCTT	0.02	174	mh16KK-049	ACTGCCCTGGAGATTGTTTCA	0.08	270
	CACTTGCGTCGTCTTTGGC				TGCTAATCCTGTCCCGTTTCT		
mh04KK-074	CCATCTTGAGTGCATTGGTTTA	0.08	172	mh16KK-096	CCGTGGACCGCTACATCTC	0.04	115
	GTTTAGCACAAGGAACCACTGAA				GTGCTGAAGACGACACTGGC		
mh05KK-022	GAGGACAGAGCCCAAACCAT	0.04	191	mh16KK-255	GGGCTTTCTGCTCAGACTTTC	0.08	236
	AGGAGACAGAAATACTCCAAGAGG				GCCTCCACGGGGACTTATTA		
mh05KK-023	TGGCACAGTGAGCACCTTCT	0.04	261	mh16KK-302	CTTATGCTTGGGTCCATCTCAG	0.04	194
	GACTTATCCCAAAGCACAAACCT				ATACCACGGATTTCCCCTCA		
mh05KK-062	AGATCACATATCATGCGACATCC	0.08	63	mh17KK-052	GCTCAGGCAGGAGGTCA	0.20	288
	TCCCTTGCTAAGTCCCTCACT				GCGCCTACTGTGCGTG		
mh05KK-078	TCAGGAAGGACAGGATAGACAGC	0.04	162	mh17KK-053	CGCTACTCTTTTGCCTGACCT	0.02	244
	AGTTCTCAGTGCCATTGCTTATC				TCCCAACTATTCTGATTCTCGC		
mh05KK-079	AAACCCTGCATATTTGCTATGG	0.08	158	mh17KK-054	CCCGCTGGAGGAGCAAAAGT	0.04	135
	GGCTCGGCGTTTTCTATTG				GAGCACGGAAGTTAGGATGGA		
mh05KK-170	GACACATGGAGGACAAAAGTGAACT	0.04	210	mh17KK-055	CCCAAAACTGACAGCCCAAG	0.20	234
	GCTGGTGATGACAAGTGAGATG				TGTGGGGTGAACAGCTCTGAC		
mh06KK-025	GGAGTTAGCCGTGGTATGTTTG	0.20	229	mh17KK-076	TCAAACCCAGAGCCATCCC	0.02	195
	CCATACGCTCCTGATAGTTGTTTA				AGGGCAAAGGACCGTGATG		
mh06KK-026	AAGGACTTTCCCTGCTGTTCTAT	0.04	158	mh17KK-077	ACAGCCTCTACCCACCAAATG	0.04	184
	ACGCAACACTCTTTTCGCTATT				AGATGTCAGCCAGAAGATCAGC		
mh06KK-080	CAGTAACACTTACTACATATGAATTGAGAA	0.20	192	mh17KK-105	CCCGTCCCTTCCAACCC	0.20	193
	CATGTCACATGTATTTTAATATCACAAA				TCTCACCTTCCCGCCTCC		
mh06KK-101	GCCTTGTAAGATTTCTCATCTGC	0.04	242	mh17KK-110	AGGTTTACCTTGGCATGTTCC	0.04	264
	AGCTGGGAGTGGCCCATG				CCAGCCCTGTTTCTAAAAGTGT		
mh07KK-030	CATTGGTAAGTTGAGTACATAACAGTTC	0.20	209	mh17KK-272	CCCTCTGGTTTTCCTTGGAT	0.20	261
	GCTTTTATGCAGTCCTAAGGAAAT				GGAACATCACGGGAATCTTTT		
mh07KK-031	GAAGGAAAGATGTCACAGATGCG	0.04	215	mh18KK-285	TCACATCATGACGTCTACTGGG	0.08	246
	GGAAAACCGCCAGCATAGC				GATCTGTTCCTCAAAGAAGAATTGG		
mh07KK-081	CCATCTGTACCACGGCATCA	0.04	245	mh18KK-293	CACCCACTGAAGTTTGAGCAGA	0.04	165
	TCTCCTACATTCATAACTCCTCCAC				CCTAATCAAGGCTATGGATACCTATCT		
mh07KK-082	AGCAGTAAAGCAGGCTGAGGC	0.08	235	mh19KK-056	CAAGCGGGAGCCCATG	0.08	289
	TTTTGGGATGTAGTGAAGAGGC				TCCCCGCCTCGGTCTC		
mh08KK-032	ACACCTCCCTGGAAACAACC	0.12	260	mh19KK-057	AAATGTCCTGGTCTTGATGGC	0.04	244
	CAACTCTTACGTTCATCAATACCG				GGGGAAAGCAGTAGTGAATGG		
mh09KK-033	TACACGGTTGCCAGAAGAAAA	0.04	175	mh19KK-299	CTCTATCATGTGGCCTGGCA	0.04	216
	GAGGTAACACTACGAGGGAAGATT				CTGGTGGGTCGCATGTCTC		
mh09KK-034	TGGTCCTGTCCTCATAGCACTT	0.12	194	mh19KK-301	TCTCAAAGACAGACCCACTACGG	0.08	168
	GTATTGAAGTGATAGTTTTACAGTTTCCTA				GAAGATTCATGCTGGCTTCAATAGT		
mh09KK-035	TTCTTTCAGCAAACCCACCC	0.04	298	mh20KK-058	TATAGAGCAGGGCCAGGCA	0.04	205
	GGCTCTGATCTGACGGCAA				GTGAAACCATCTCCAAGTCCAG		
mh09KK-152	AATGTGGTAACTGAGACTAGGAGAATC	0.08	241	mh20KK-059	TCATAGCAGCTGGTCTCGTTG	0.04	225
	TCGAACTTCATAGGCTGACTCC				TCCCTGGCTGTGCTCATGT		
mh09KK-153	GGGGATTGGCAGTCTTCATG	0.04	180	mh20KK-307	TCCTACAGCATTCAATTACCAAAGC	0.08	250
	ACAGCCTCGTAAGGGGAGCT				TGAGCATTACCACGATCACTTCTA		
mh09KK-157	AGTCTAGGGCTGGAGTTGGGT	0.04	233	mh21KK-315	TTATGTGGTAGGAGCCTAAAAGAAG	0.04	285
	GGACCATCAGCATCAATAGCC				TGTGACCCCTGACCTTGCTG		
mh10KK-083	GTGGTTCTATTTAATGTGAAGCCTG	0.04	224	mh21KK-316	TCATAAACTACAGCTGGCAGACC	0.04	208
	GCTGGCAGAACTGGGATTTG				CTCCTTAATATCTTCCCATGTCCA		
mh10KK-084	CTGTTGCTAATATCTTACCTGCTCC	0.02	126	mh21KK-320	TGACTGGGAGGCTGTGGAGA	0.04	283
	GCTCTTACACGAAGTTACATTAGGGA				TGCTGGAATTAGAGGCGTGA		
mh10KK-085	AAGGGGCAGAAACTGGGAG	0.04	117	mh21KK-324	GGGCGAGCAGGGGTCA	0.04	196
	GGGGATGGAAAACAGAGCC				GCATTTCCGCTGACGCTAT		
mh10KK-086	TGGATTGGAGCCCAGGTATT	0.08	167	mh22KK-060	CGTGATTCAGGAGCACCAGC	0.08	213
	ACACTGATTTCCCTCAAGGTCA				TTTTCCAGGTCTGACAACGG		
mh10KK-087	AAAGACTTGCTCCATTCCCTATTC	0.12	231	mh22KK-061	CTTTAGGGGTGGCAAGTCTCC	0.02	218
	TGATTCTCCACGCTGCCA				CCACTTAGGGACTGGGGAACTC		
mh10KK-088	CAAAACTACATTCTTCACTGGGG	0.08	250	mh22KK-064	AAAGCGGTGAACAGGTGGA	0.04	263
	ACTGCCTCTGATCTTTCTCACCT				TGGTCACAGTTCTTGGTCCG		
mh10KK-101	CCCAGGACTGTCTGAGCATCT	0.02	170	mh22KK-069	GCAGCACTTTCTTTCATTCATTCC	0.04	144
	TGTCTCCCTCCACAGCATGA				AACCATGAGTGCTACAAAGGC		
mh11KK-036	GCCAAAGCTCCCTAATAGCTC	0.08	240	mh22KK-303	AGTTCATCCTGCAGCCCATC	0.02	181
	CAGAAATAAAAGGCTAAATGTATGGAT				CGGACCCCACCTTTCTTGT		

C: Final concentrations of the primers; AS: Amplicon sizes.

**Figure 1:**
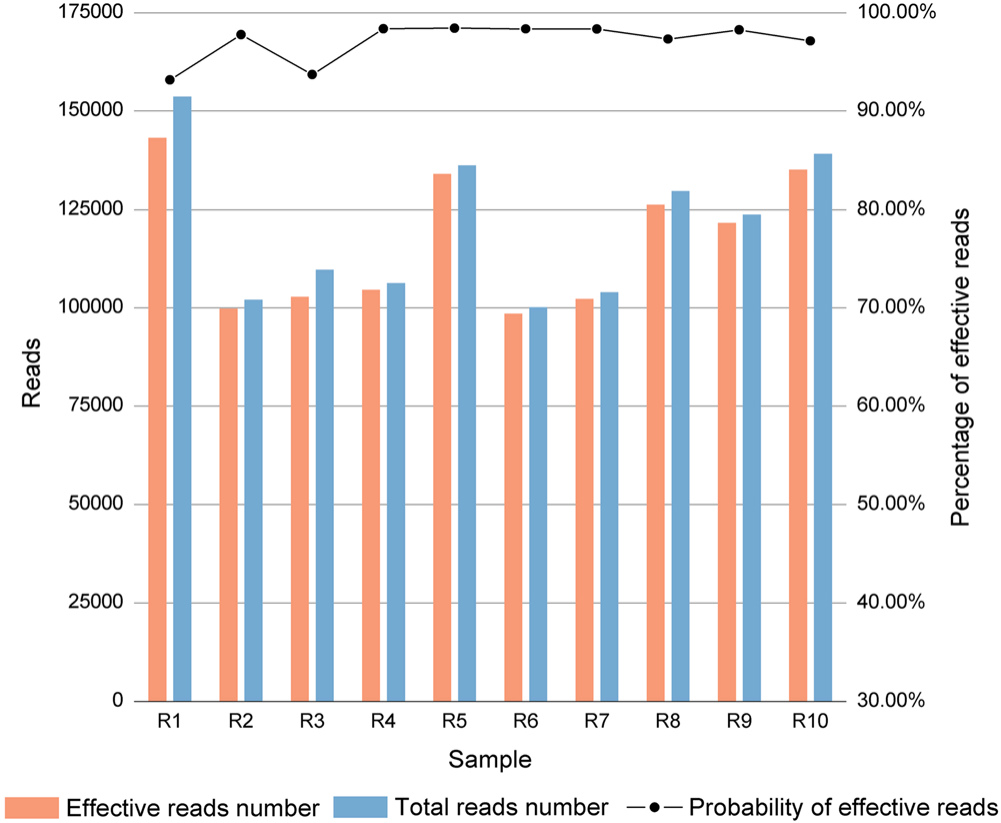
Read counts and percentage of reads representing the alleles for 10 reference samples. Number of effective reads (those called as microhaplotype alleles) are shown in orange, and the total reads are shown in blue.

To evaluate the performance of this assay, we sequenced 10 reference samples. The numbers of total reads and reads representing microhaplotype alleles were calculated and are shown in Fig. 1. Around 100,000 total reads were obtained for each sample. The reads representing alleles accounted for over 90% of the total reads, and even 99% for some samples, indicating that the quality of the sequencing data was good.

**Figure 2:**
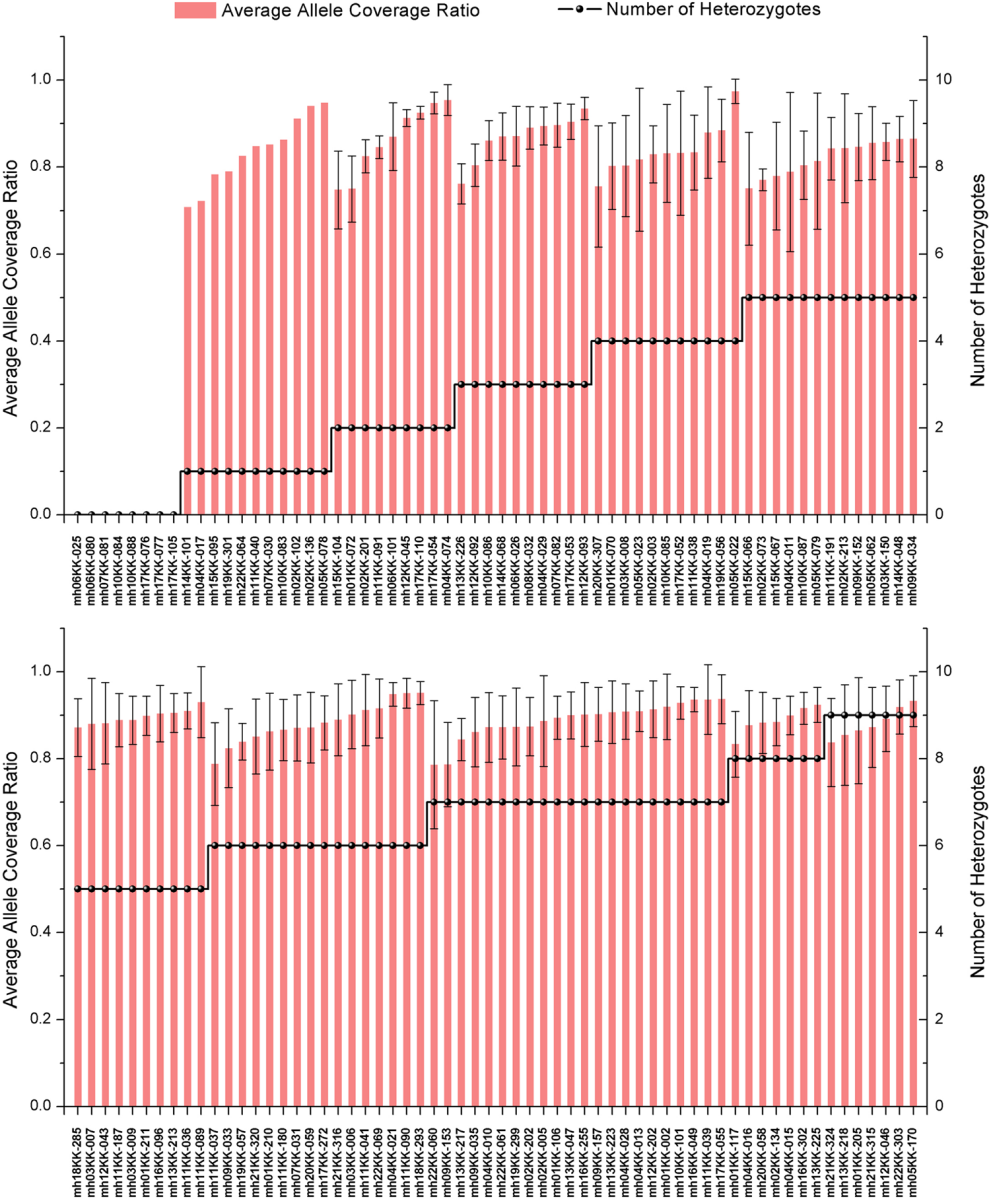
Average allele coverage ratio (ACR) for each locus. Horizontal black line, number of heterozygotes for each calculated ACR. Error bars represent standard deviations.

**Figure 3:**
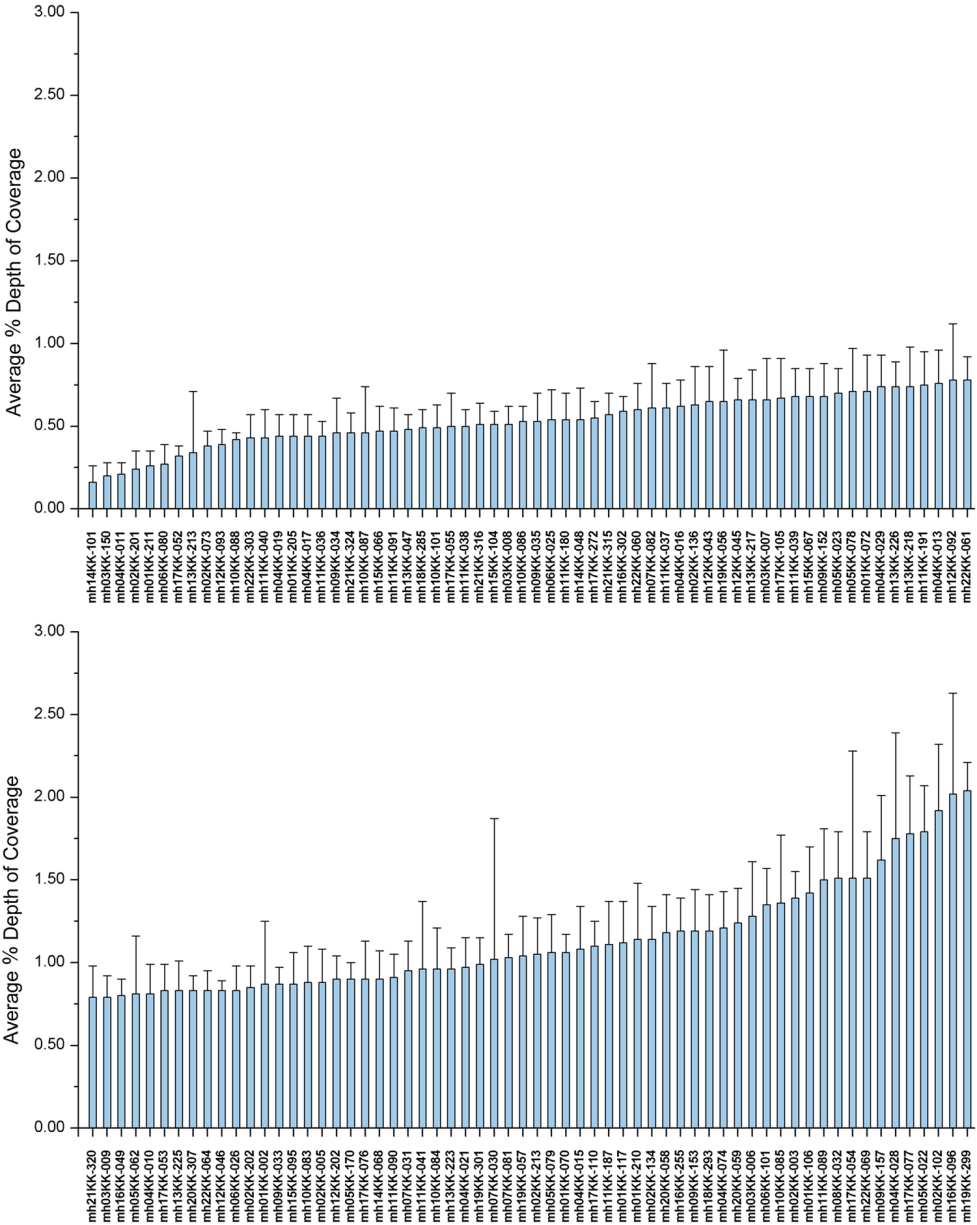
Average percentage (%) depth of coverage (DoC) for each locus. Error bars represent standard deviations.

The allele coverage ratio (ACR) was used to evaluate the heterozygosity balance. The ACRs were calculated for the 10 reference samples by dividing the lower coverage allele by the higher coverage allele at each locus. All average ACRs were above 0.7, indicating that the heterozygosity balance of the 124-plex assay was good (Fig. 2). To examine the interlocus balance of this 124-plex panel, we calculated the average percentage depth of coverage (DoC) for each locus (Fig. 3). Each locus accounted for 0.2%–2% of the effective reads, 0.8% on average.

To evaluate the sensitivity of the 124-plex assay, a dilution series of genomic DNA 9947 A (1.0, 0.5, 0.2, and 0.1 ng) was sequenced. All 124 microhaplotypes were successfully genotyped with a sequencing depth of ≥ 30 × when 1.0 ng, 0.5 ng, 0.2 ng, or 0.1 ng of input DNA was used (Supplementary Table S2 and Supplementary Figs. S2–S5), demonstrating the highly sensitive performance of the 124-plex assay.

### Mixture study

To compare the effectiveness of microhaplotypes and STRs in the analysis of forensic mixtures, we prepared artificially mixed DNA samples with commercial genomic DNAs 9947 A and 2800 M, and performed parallel CE-based STR profiling and NGS-based microhaplotype genotyping experiments (Table 2 and Supplementary Figs. S5–S18). Representative data are summarized and compared in Fig. 4.

**Table 2:** Summary of STR-based and microhaplotype-based analysis of artificially mixed biological samples.

Genetic marker	Mixtures	Number of loci with fully called 9947 A alleles	9947 A drop-out loci	Number of loci interfered by stutters in mixture deconvolution	Loci interfered by stutters in mixture deconvolution	Number of remaining effective loci	Percentage of remaining effective loci
STR	9947 A:2800 M = 1:1	21		0		21	100.00%
	9947 A**:**2800 M = 1**:**3	21		0		21	100.00%
	9947 A**:**2800 M = 1**:**6	21		7	D16S539, CSF1PO, D18S51, D19S433, FGA, D22S1045, D2S1338	14	66.67%
	9947 A**:**2800 M = 1**:**9	19	D22S1045, D2S1338	11	D3S1358, vWA, D16S539, CSF1PO, D18S51, D19S433, FGA, D5S818, D7S820, D10S1248, D12S391	8	38.10%
	9947 A**:**2800 M = 1**:**19	8	D3S1358, vWA, D16S539, CSF1PO, TPOX, D18S51, D19S433, TH01, D22S1045, SE33, D1S1656, D12S391, D2S1338	7	D8S1179, D21S11, FGA, D5S818, D13S317, D7S820, D10S1248	1	4.76%
Microhaplotype	9947 A:2800 M = 1:1	124		0		124	100.00%
	9947 A**:**2800 M = 1**:**3	123	mh02KK-136	0		123	99.19%
	9947 A**:**2800 M = 1**:**6	123	mh02KK-136	0		123	99.19%
	9947 A**:**2800 M = 1**:**9	123	mh02KK-136	0		123	99.19%
	9947 A**:**2800 M = 1**:**19	114	mh01KK-205, mh02KK-136, mh04KK-019, mh05KK-079, mh07KK-030, mh08KK-032, mh10KK-087, mh12KK-043, mh17KK-055, mh21KK-324	0		114	91.94%

**Figure 4:**
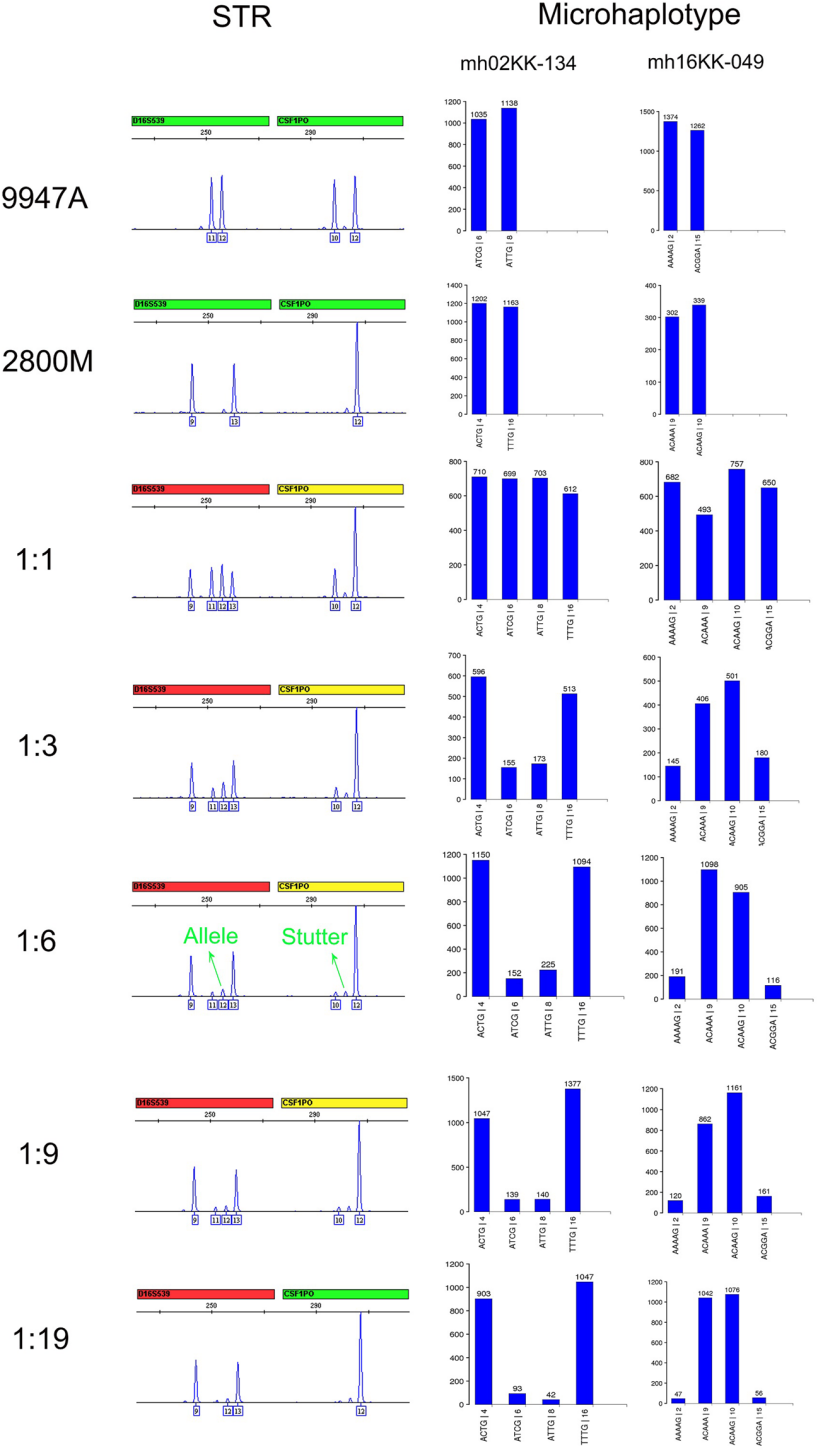
Representative STR profiles and representative microhaplotype genotyping histograms for the mixture experiments. Signal peaks for D16S539-12 and CSF1PO-11 in the 1:6 mixture are indicated as “Allele” and “Stutter”, respectively, for comparison. Numbers under each microhaplotype allele are the numeral allele names assigned to allow the microhaplotype data to be read conveniently.

Allele dropouts can severely interfere with a mixture analysis. Therefore, we examined the dropout alleles of the minor contributor (9947 A), and calculated the number of loci with fully called 9947 A alleles for each artificially mixed sample. In the STR profiles, no allele dropout was observed for the 1:1, 1:3, or 1:6 mixture (Table 2). Two alleles (D22S1045-11 and D2S1338-19) of the minor contributor dropped out in the analysis of the 1:9 mixture (Supplementary Fig. S12), and only 38% of the STR loci (8/21) reported full 9947 A alleles when the mixture ratio was 1:19 (Table 2, Supplementary Fig. S13). In contrast, 92% of the microhaplotypes (114/124) reported full 9947 A alleles for the 1:19 mixture (Table 2, Supplementary Fig. S18). No allele dropin was observed for the 1:3, 1:6, 1:9, or 1:19 mixture. Only two artefacts dropped in (mh02KK003-GTC and mh20kk059-AG) with low sequencing depths (40X and 30X, see Supplementary Fig. S19) when analyzing the 1:1 mixture. These data indicated that the NGS-based microhaplotypes were superior to the CE-based STRs in genotyping the alleles of the minor contributor.

We then investigated the effect of STR stutters on the analysis of these mixtures. When 9947 A and 2800 M were mixed at a 1:1 ratio, the alleles from both contributors were very similar in peak height or sequencing depth (Fig. 4, Supplementary Figs. S9 and S14). Neither STR nor microhaplotype was effective in mixture deconvolution. When the mixture ratio was 1:3, the peak heights of alleles from the minor contributor were significantly lower than those of the major contributor and significantly higher than the STR stutters (Fig. 4, Supplementary Figs. S10 and S15). Both STRs and microhaplotypes were effective in mixture deconvolution. However, at mixture ratios of 1:6, 1:9, and 1:19, the minor contributor STR alleles were indistinguishable from the stutters of the major contributor because their peak heights were similar (Fig. 4). For example, in the 1:6 mixture, D16S539-12 (an allele of the minor contributor 9947 A) and CSF1PO-11 (a stutter of the major contributor 2800 M) were both at the n-1 stutter position, with similar intensities. Their peak heights were 5%−10% of those of their possible parent alleles, which is typical for STR stutters. Incorrect allele/stutter interpretation can readily occur in such situations. However, with microhaplotype genotyping, the alleles from the major and minor contributors were easily distinguishable in the various mixture ratios based on their sequencing depths (Table 2, Supplementary Figs. S15–S18). Taken together, only 38.10% and 4.76% of the STR loci were effective in analyzing the 1:9 and 1:19 mixtures, respectively, whereas 99.19% and 91.94% of the microhaplotypes were effective in analyzing the same mixtures, respectively (Table 2). These data confirm that microhaplotypes are reliable genetic markers for the deconvolution of forensic mixtures.

### Population data

A total of 256 Han Chinese individuals residing in Gansu Province were genotyped, and 514 alleles were observed (Table 3), with approximately four alleles per locus on average. Thirteen alleles were observed for locus mh01KK-117, which was the highest number in this dataset. Single alleles were observed for two loci, mh10KK-084 and mh17KK-076, indicating that there was no genetic diversity at these two loci in this Han Chinese population. Therefore, the forensic parameters were not calculated for these two loci. The forensic statistical parameters were calculated for the other 122 loci, and are summarized in Table 4.

**Table 3:** Allele frequencies of 124 microhaplotypes in the Chinese Han population (N = 256).

Genot	Count	Fre	Genot	Count	Fre	Genot	Count	Fre	Genot	Count	Fre
mh01KK-002			mh01KK-070			mh01KK-072			mh01KK-106		
AA	323	0.6333	AG	97	0.1895	CG	351	0.6937	CAAG	1	0.0022
AG	120	0.2353	AT	415	0.8105	TC	155	0.3063	CAGA	251	0.5553
GA	19	0.0373	mh01KK-205			mh01KK-210			CAGG	68	0.1504
GG	48	0.0941	CCAG	130	0.2632	CC	82	0.1660	CGAG	5	0.0111
mh01KK-117			TCAG	87	0.1761	TC	143	0.2895	TAGG	127	0.2810
AACC	193	0.3955	TTAA	74	0.1498	TT	269	0.5445	mh01KK-211		
AACT	84	0.1721	TTAG	66	0.1336	mh02KK-073			ACT	30	0.0673
AAGC	12	0.0246	TTGG	137	0.2773	GC	358	0.7075	ATC	193	0.4327
AAGT	6	0.0123	mh02KK-005			GT	105	0.2075	ATT	127	0.2848
AGCC	54	0.1107	AG	195	0.3916	TC	2	0.0040	GCC	3	0.0067
AGCT	15	0.0307	GA	174	0.3494	TT	41	0.0810	GTC	93	0.2085
AGGC	3	0.0061	GG	129	0.2590	mh02KK-201			mh02KK-102		
CACC	83	0.1701	mh02KK-136			GA	14	0.0287	GAC	11	0.0259
CACT	5	0.0102	GTA	4	0.0116	GG	22	0.0451	GGT	409	0.9646
CAGC	15	0.0307	GTC	5	0.0145	TA	452	0.9262	TGC	4	0.0094
CAGT	1	0.0020	TCA	11	0.0320	mh03KK-007			mh02KK-202		
CGCC	8	0.0164	TCC	185	0.5378	CC	184	0.3622	CA	253	0.4961
CGCT	9	0.0184	TTC	139	0.4041	TC	183	0.3602	CC	1	0.0020
mh02KK-003			mh03KK-006			TT	141	0.2776	GA	256	0.5020
GCC	2	0.0039	AA	288	0.5692	mh03KK-150			mh03KK-008		
GTC	18	0.0353	AG	169	0.3340	AACA	162	0.3476	CG	1	0.0020
TCC	396	0.7765	TA	49	0.0968	GACC	191	0.4099	CT	21	0.0427
TTC	48	0.0941	mh03KK-009			GGCC	113	0.2425	TG	258	0.5244
TTT	46	0.0902	CC	19	0.0373	mh04KK-010			TT	212	0.4309
mh02KK-134			TC	144	0.2824	AA	292	0.5703	mh04KK-011		
ACCG	23	0.0456	TT	347	0.6804	AG	166	0.3242	AC	215	0.4674
ACTA	3	0.0060	mh04KK-013			GA	47	0.0918	AT	118	0.2565
ACTG	25	0.0496	AAGAT	48	0.0964	GG	7	0.0137	GT	127	0.2761
ATCA	19	0.0377	CAGAT	61	0.1225	mh04KK-015			mh04KK-016		
ATCG	234	0.4643	CAGGT	3	0.0060	AC	339	0.6673	CC	60	0.1172
ATTA	56	0.1111	CGAAT	2	0.0040	AT	112	0.2205	TC	116	0.2266
ATTG	56	0.1111	CGGAC	27	0.0542	TT	57	0.1122	TT	336	0.6563
TCTA	1	0.0020	CGGAT	64	0.1285	mh04KK-017			mh04KK-029		
TCTG	11	0.0218	CGGGT	293	0.5884	ACA	25	0.0912	TC	422	0.8242
TTCG	7	0.0139	mh04KK-021			GCA	18	0.0657	TT	90	0.1758
TTTG	69	0.1369	AG	160	0.3226	GCG	185	0.6752	mh05KK-023		
mh02KK-213			GA	162	0.3266	GTA	46	0.1679	GCG	26	0.0544
CAT	29	0.0566	GG	174	0.3508	mh04KK-028			TCG	297	0.6213
CGT	156	0.3047	mh05KK-022			CA	2	0.0039	TTG	140	0.2929
TGC	17	0.0332	CA	259	0.5078	CC	156	0.3047	TTT	15	0.0314
TGT	310	0.6055	CC	152	0.2980	TC	354	0.6914	mh05KK-062		
mh04KK-019			TC	99	0.1941	mh05KK-170			AA	136	0.2677
AA	226	0.4431	mh05KK-079			CAAA	49	0.0984	AC	248	0.4882
AG	262	0.5137	CC	275	0.5413	CAAG	50	0.1004	TA	124	0.2441
GA	22	0.0431	CT	233	0.4587	CAGA	12	0.0241	mh06KK-025		
mh04KK-074			mh06KK-080			CAGG	14	0.0281	AGG	43	0.1503
AC	1	0.0020	AG	8	0.0158	CGAA	63	0.1265	GGG	243	0.8497
AT	446	0.8745	CG	498	0.9842	CGAG	60	0.1205	mh07KK-030		
GT	63	0.1235	mh07KK-081			CGGA	35	0.0703	ACC	159	0.6023
mh05KK-078			-C	3	0.0059	CGGG	16	0.0321	GAC	52	0.1970
GA	81	0.1582	-T	509	0.9941	TAAA	74	0.1486	GCC	53	0.2008
GG	431	0.8418	mh09KK-034			TAAG	123	0.2470	mh08KK-032		
mh06KK-026			AA	14	0.0276	TAGG	1	0.0020	CG	67	0.1683
ACG	1	0.0020	GA	108	0.2126	TGAA	1	0.0020	TA	39	0.0980
ATG	17	0.0335	GG	386	0.7598	mh06KK-101			TG	292	0.7337
GCA	12	0.0236	mh09KK-153			AA	413	0.8381	mh09KK-152		
GCG	461	0.9075	CAA	23	0.0477	GA	1	0.0020	AGCA	97	0.1964
GTG	17	0.0335	CAC	31	0.0643	GG	79	0.1599	ATCG	1	0.0020
mh07KK-031			CGA	5	0.0104	mh07KK-082			ATTA	31	0.0628
CA	269	0.5316	TAA	191	0.3963	TC	198	0.3898	ATTG	267	0.5405
CG	98	0.1937	TAC	116	0.2407	TG	310	0.6102	GTCG	98	0.1984
TG	139	0.2747	TGA	87	0.1805				mh10KK-083		
mh09KK-033			TGC	29	0.0602	mh09KK-035			GC	41	0.0807
ACG	164	0.3241	mh09KK-157			CG	157	0.3257	TC	467	0.9193
GCG	181	0.3577	ACCAT	15	0.0305	CT	195	0.4046	mh10KK-088		
GCT	8	0.0158	ACTAT	45	0.0915	TG	130	0.2697	GC	355	0.9492
GTG	153	0.3024	GCCAC	239	0.4858	mh10KK-087			GT	19	0.0508
mh10KK-084			GCCCC	2	0.0041	AG	351	0.6964	mh11KK-038		
TG	512	1.0000	GCCCT	139	0.2825	GA	153	0.3036	CG	236	0.5388
mh10KK-085			GTCAC	52	0.1057	mh11KK-037			TA	18	0.0411
CC	254	0.4961	mh10KK-086			ACG	202	0.4040	TG	184	0.4201
CT	258	0.5039	GA	302	0.5945	GCG	208	0.4160	mh11KK-089		
mh10KK-101			GC	149	0.2933	GTG	90	0.1800	AT	264	0.5156
AG	181	0.3620	TA	57	0.1122	mh11KK-041			CG	219	0.4277
CA	80	0.1600	mh11KK-036			AG	45	0.0893	CT	29	0.0566
CG	239	0.4780	AA	150	0.2941	GA	282	0.5595	mh11KK-187		
mh11KK-039			AG	155	0.3039	GG	177	0.3512	CCCA	226	0.4575
GG	36	0.0706	CG	205	0.4020	mh11KK-180			CCCG	6	0.0121
GT	221	0.4333	mh11KK-040			AACC	3	0.0066	GCCG	3	0.0061
TT	253	0.4961	AC	308	0.8324	AACG	1	0.0022	GCGA	2	0.0040
mh11KK-090			CG	62	0.1676	AATC	38	0.0830	GCGG	133	0.2692
AC	331	0.6490	mh11KK-091			AATG	1	0.0022	GTCA	1	0.0020
GT	179	0.3510	-C	78	0.1535	ACCC	200	0.4367	GTGG	123	0.2490
mh11KK-191			-T	430	0.8465	ACCG	13	0.0284	mh12KK-046		
CAGT	103	0.2239	mh12KK-043			ACTC	54	0.1179	GA	144	0.2824
CGAT	65	0.1413	CCG	47	0.0925	ACTG	11	0.0240	GG	130	0.2549
TAAC	86	0.1870	CTA	251	0.4941	GCCC	5	0.0109	TA	133	0.2608
TAAT	204	0.4435	CTG	209	0.4114	GCCG	128	0.2795	TG	103	0.2020
TGAT	2	0.0043	TCG	1	0.0020	GCTC	3	0.0066	mh13KK-047		
mh12KK-092			mh12KK-093			GCTG	1	0.0022	CC	103	0.2146
CT	183	0.3735	AT	402	0.7882	mh12KK-045			CT	73	0.1521
TC	307	0.6265	TA	108	0.2118	CT	35	0.0694	TC	15	0.0313
mh13KK-213			mh13KK-217			TC	394	0.7817	TT	289	0.6021
CCA	97	0.3255	AACA	6	0.0121	TT	75	0.1488	mh13KK-223		
CCG	54	0.1812	AACG	26	0.0526	mh12KK-202			CCCT	79	0.1561
TAG	51	0.1711	AATA	1	0.0020	AACT	97	0.1972	CGCC	1	0.0020
TCA	83	0.2785	AATG	178	0.3603	AATC	181	0.3679	CGCT	104	0.2055
TCG	13	0.0436	AGCA	61	0.1235	AGTT	92	0.1870	CGTC	139	0.2747
mh13KK-218			AGCG	66	0.1336	CATC	2	0.0041	CGTT	100	0.1976
CCCC	8	0.0161	AGTG	80	0.1619	CATT	119	0.2419	TCCT	16	0.0316
CCCT	20	0.0403	GATG	1	0.0020	CGTT	1	0.0020	TGCT	67	0.1324
CTCC	47	0.0948	GGCA	2	0.0040	mh14KK-048			mh14KK-068		
CTCT	61	0.1230	GGCG	67	0.1356	AC	7	0.0152	AC	183	0.3574
CTTC	95	0.1915	GGTG	6	0.0121	AT	273	0.5935	AT	286	0.5586
CTTT	48	0.0968	mh13KK-226			GC	37	0.0804	CC	43	0.0840
TCCC	2	0.0040	CA	10	0.0207	GT	143	0.3109	mh15KK-067		
TCCT	4	0.0081	CG	138	0.2851	mh15KK-066			GC	230	0.4563
TTCC	18	0.0363	TA	336	0.6942	AG	202	0.4139	GT	88	0.1746
TTCT	73	0.1472	mh14KK-101			AT	62	0.1270	TC	176	0.3492
TTTC	22	0.0444	AT	71	0.1530	CG	115	0.2357	TT	10	0.0198
TTTT	98	0.1976	GC	13	0.0280	CT	109	0.2234	mh16KK-255		
mh13KK-225			GT	380	0.8190	mh16KK-096			ACCG	38	0.0769
AAG	65	0.1280	mh15KK-095			CA	328	0.6457	ACTA	1	0.0020
ACG	200	0.3937	CA	260	0.5078	CG	179	0.3524	ACTG	155	0.3138
GAA	103	0.2028	TA	221	0.4316	TG	1	0.0020	GACA	173	0.3502
GAG	133	0.2618	TG	31	0.0605	mh16KK-302			GATA	17	0.0344
GCG	7	0.0138	mh16KK-049			ACTT	62	0.1225	GCCA	4	0.0081
mh15KK-104			AAAAG	150	0.3036	GCTC	99	0.1957	GCCG	39	0.0789
CAG	8	0.0158	ACAAA	43	0.0870	GCTT	81	0.1601	GCTG	67	0.1356
TAA	8	0.0158	ACAAG	5	0.0101	GTAT	188	0.3715	mh17KK-054		
TAG	54	0.1067	ACAGA	12	0.0243	GTTT	76	0.1502	AA	183	0.4816
TCG	436	0.8617	ACGGA	211	0.4271	mh17KK-053			AG	84	0.2211
mh17KK-055			CCAAA	72	0.1457	CT	234	0.4699	GG	113	0.2974
AC	227	0.5881	CCGGA	1	0.0020	TC	205	0.4116	mh17KK-105		
AT	1	0.0026	mh17KK-052			TT	59	0.1185	ATA	12	0.0235
CC	47	0.1218	AA	101	0.2186	mh17KK-077			ATG	498	0.9765
CT	111	0.2876	AG	148	0.3203	GG	440	0.8594	mh18KK-293		
mh17KK-110			GA	202	0.4372	TG	72	0.1406	AGAA	121	0.2430
CA	7	0.0137	GG	11	0.0238	mh18KK-285			AGGA	1	0.0020
CG	432	0.8471	mh17KK-076			AGCG	45	0.0886	ATAA	8	0.0161
TG	71	0.1392	AG	512	1.0000	CACG	256	0.5039	ATGA	72	0.1446
mh19KK-056			mh17KK-272			CGCG	6	0.0118	GGAA	198	0.3976
CA	265	0.5430	CCCT	246	0.5125	CGCT	89	0.1752	GGAG	77	0.1546
CC	1	0.0020	TCAT	25	0.0521	CGTG	112	0.2205	GGGA	9	0.0181
TA	18	0.0369	TCCC	28	0.0583	mh19KK-299			GTAA	7	0.0141
TC	204	0.4180	TCCT	116	0.2417	ACGAA	1	0.0020	GTAG	2	0.0040
mh21KK-315			TTCC	65	0.1354	ATGAA	68	0.1382	GTGA	3	0.0060
ACC	28	0.0562	mh19KK-057			ATGAG	1	0.0020	mh19KK-301		
ACT	1	0.0020	CCG	331	0.6567	GCAAA	50	0.1016	AGGT	4	0.0078
ATC	106	0.2129	CTG	139	0.2758	GCAAG	219	0.4451	GAAC	416	0.8157
ATT	13	0.0261	CTT	34	0.0675	GCATG	100	0.2033	GGAC	2	0.0039
GCC	69	0.1386	mh20KK-059			GCGTA	43	0.0874	GGAT	88	0.1725
GCT	28	0.0562	AA	136	0.2698	GCGTG	10	0.0203	mh20KK-058		
GTC	84	0.1687	AG	50	0.0992	mh20KK-307			CAC	162	0.3240
GTT	169	0.3394	GG	318	0.6310	CTGA	142	0.2971	TAC	148	0.2960
mh21KK-316			mh21KK-320			TTAA	101	0.2113	TAT	136	0.2720
ACAC	198	0.3976	AACA	56	0.1181	TTGA	192	0.4017	TGC	54	0.1080
ACGC	3	0.0060	AACG	127	0.2679	TTGC	43	0.0900	mh22KK-060		
ACGT	132	0.2651	AATA	1	0.0021	mh21KK-324			CA	144	0.2903
ATGC	44	0.0884	AGCG	1	0.0021	CCAA	3	0.0062	CG	170	0.3427
GCGC	120	0.2410	GACA	141	0.2975	CCAG	14	0.0288	GG	182	0.3669
GTGC	1	0.0020	GACG	18	0.0380	CCTA	19	0.0391	mh22KK-303		
mh22KK-061			GATA	81	0.1709	CCTG	3	0.0062	CGGG	325	0.6423
AAA	84	0.1667	GGCA	22	0.0464	CTAA	140	0.2881	CTGG	36	0.0711
AAG	4	0.0079	GGCG	27	0.0570	CTTA	50	0.1029	TGGG	145	0.2866
GAA	256	0.5079	mh22KK-064			CTTG	1	0.0021	mh22KK-069		
GAG	31	0.0615	AAT	432	0.8438	TCAG	185	0.3807	AG	46	0.0898
GGG	129	0.2560	GAT	80	0.1563	TCTG	69	0.1420	GG	166	0.3242
						TTAA	2	0.0041	GT	300	0.5859

Genot: allele genotype; Count: allele count; Fre: allele frequency.

**Table 4:** Forensic parameters of 122 microhaplotypes in the Chinese Han population (N = 256).

Microhaplotype	MP	PD	PE	TPI	H_o_	H_e_	p	A_e_
mh01KK-002	0.2706	0.7294	0.2261	1.0897	0.5412	0.5343	0.5217	2.1426
mh01KK-070	0.5323	0.4677	0.0518	0.6845	0.2695	0.3077	0.0636	1.4433
mh01KK-072	0.4226	0.5774	0.1338	0.8785	0.4308	0.4258	0.8834	1.7391
mh01KK-106	0.2293	0.7707	0.1954	1.0180	0.5089	0.5912	0.0002	2.4386
mh01KK-117	0.0858	0.9142	0.5455	2.1786	0.7705	0.7710	0.3833	4.3362
mh01KK-205	0.0826	0.9174	0.5504	2.2054	0.7733	0.7841	0.5912	4.5984
mh01KK-210	0.2356	0.7644	0.2485	1.1435	0.5628	0.5933	0.4856	2.4518
mh01KK-211	0.1476	0.8524	0.2813	1.2253	0.5919	0.6851	0.0600	3.1606
mh02KK-003	0.4045	0.5955	0.0981	0.7969	0.3726	0.3796	0.8549	1.6099
mh02KK-005	0.1928	0.8072	0.3729	1.4821	0.6627	0.6588	0.9792	2.9197
mh02KK-073	0.3620	0.6380	0.1338	0.8785	0.4308	0.4507	0.0288	1.8175
mh02KK-102	0.8745	0.1255	0.0034	0.5327	0.0613	0.0689	0.2225	1.0738
mh02KK-134	0.0952	0.9048	0.5098	2.0000	0.7500	0.7358	0.8278	3.7641
mh02KK-136	0.3507	0.6493	0.0166	0.5850	0.1454	0.5477	0.0000	2.2033
mh02KK-201	0.7437	0.2563	0.0154	0.5810	0.1393	0.1395	0.5707	1.1618
mh02KK-202	0.3596	0.6404	0.1662	0.9515	0.4745	0.5029	0.3858	2.0078
mh02KK-213	0.2878	0.7122	0.2441	1.1327	0.5586	0.5373	0.9422	2.1564
mh03KK-006	0.2830	0.7170	0.2428	1.1295	0.5573	0.5562	0.6474	2.2478
mh03KK-007	0.1898	0.8102	0.3767	1.4941	0.6654	0.6633	0.7868	2.9586
mh03KK-008	0.3124	0.6876	0.1840	0.9919	0.4959	0.5386	0.0204	2.1623
mh03KK-009	0.3660	0.6340	0.1396	0.8916	0.4392	0.4569	0.7746	1.8381
mh03KK-150	0.1785	0.8215	0.1894	1.0043	0.5022	0.6538	0.0000	2.8765
mh04KK-010	0.2778	0.7222	0.3023	1.2800	0.6094	0.5621	0.3058	2.2780
mh04KK-011	0.2048	0.7952	0.2857	1.2366	0.5957	0.6409	0.0016	2.7741
mh04KK-013	0.1898	0.8102	0.2748	1.2087	0.5864	0.6113	0.0178	2.5644
mh04KK-015	0.3147	0.6853	0.1945	1.0160	0.5079	0.4945	0.8615	1.9743
mh04KK-016	0.3061	0.6939	0.2015	1.0323	0.5156	0.5053	0.8547	2.0172
mh04KK-017	0.4434	0.5566	0.0330	0.6343	0.2117	0.5052	0.0000	2.0133
mh04KK-019	0.3128	0.6872	0.1927	1.0119	0.5059	0.5389	0.1373	2.1638
mh04KK-021	0.1854	0.8146	0.3378	1.3778	0.6371	0.6676	0.0402	2.9958
mh04KK-028	0.4162	0.5838	0.1384	0.8889	0.4375	0.4300	0.9446	1.7516
mh04KK-029	0.5456	0.4544	0.0623	0.7111	0.2969	0.2903	0.8304	1.4080
mh04KK-074	0.6374	0.3626	0.0308	0.6281	0.2039	0.2204	0.3388	1.2820
mh05KK-022	0.2202	0.7798	0.3201	1.3281	0.6235	0.6168	0.8422	2.6014
mh05KK-023	0.2921	0.7079	0.1893	1.0042	0.5021	0.5253	0.7528	2.1018
mh05KK-062	0.2033	0.7967	0.2843	1.2330	0.5945	0.6317	0.4003	2.7058
mh05KK-078	0.5705	0.4295	0.0576	0.6995	0.2852	0.2669	0.3472	1.3630
mh05KK-079	0.3957	0.6043	0.2166	1.0672	0.5315	0.4976	0.3140	1.9864
mh05KK-170	0.0380	0.9620	0.7619	4.2931	0.8835	0.8610	0.4205	7.1065
mh06KK-025	0.7376	0.2624	0.0000	0.5035	0.0070	0.2564	0.0000	1.3432
mh06KK-026	0.6922	0.3078	0.0229	0.6048	0.1732	0.1740	0.3517	1.2102
mh06KK-080	0.9534	0.0466	0.0002	0.5080	0.0158	0.0312	0.0008	1.0321
mh06KK-101	0.5701	0.4299	0.0453	0.6676	0.2510	0.2726	0.3590	1.3738
mh07KK-030	0.3246	0.6754	0.0617	0.7097	0.2955	0.5603	0.0000	2.2633
mh07KK-031	0.2342	0.7658	0.3316	1.3602	0.6324	0.6056	0.3496	2.5279
mh07KK-081	0.9768	0.0232	0.0001	0.5059	0.0117	0.0117	1.0000	1.0118
mh07KK-082	0.3776	0.6224	0.1465	0.9071	0.4488	0.4766	0.3554	1.9073
mh08KK-032	0.4155	0.5845	0.0488	0.6769	0.2613	0.4249	0.0000	1.7355
mh09KK-033	0.1789	0.8211	0.3805	1.5060	0.6680	0.6767	0.0061	3.0799
mh09KK-034	0.4537	0.5463	0.0788	0.7515	0.3347	0.3774	0.0003	1.6043
mh09KK-035	0.1928	0.8072	0.3572	1.4345	0.6515	0.6589	0.6051	2.9196
mh09KK-152	0.1894	0.8106	0.2948	1.2602	0.6032	0.6273	0.4162	2.6740
mh09KK-153	0.1031	0.8969	0.4174	1.6284	0.6930	0.7439	0.1148	3.8810
mh09KK-157	0.1652	0.8348	0.3728	1.4819	0.6626	0.6651	0.2222	2.9738
mh10KK-083	0.7378	0.2622	0.0167	0.5853	0.1457	0.1487	0.6690	1.1742
mh10KK-085	0.3750	0.6250	0.1875	1.0000	0.5000	0.5010	1.0000	1.9999
mh10KK-086	0.2699	0.7301	0.2204	1.0763	0.5354	0.5491	0.3924	2.2122
mh10KK-087	0.4464	0.5536	0.1773	0.9767	0.4881	0.4237	0.0178	1.7326
mh10KK-088	0.8779	0.1221	0.0007	0.5137	0.0267	0.0967	0.0000	1.1067
mh10KK-101	0.2269	0.7731	0.3055	1.2887	0.6120	0.6161	0.9879	2.5965
mh11KK-036	0.1919	0.8081	0.3730	1.4826	0.6628	0.6609	0.7357	2.9373
mh11KK-037	0.2118	0.7882	0.3417	1.3889	0.6400	0.6326	0.0278	2.7124
mh11KK-038	0.3318	0.6682	0.2148	1.0631	0.5297	0.5327	0.0017	2.1345
mh11KK-039	0.3052	0.6948	0.2725	1.2028	0.5843	0.5623	0.1308	2.2787
mh11KK-040	0.6233	0.3767	0.0115	0.5675	0.1189	0.2797	0.0000	1.3869
mh11KK-041	0.2801	0.7199	0.1981	1.0244	0.5119	0.5567	0.0569	2.2504
mh11KK-089	0.2959	0.7041	0.2202	1.0756	0.5352	0.5490	0.8886	2.2122
mh11KK-090	0.4014	0.5986	0.1539	0.9239	0.4588	0.4565	1.0000	1.8368
mh11KK-091	0.5853	0.4147	0.0430	0.6615	0.2441	0.2605	0.3349	1.3512
mh11KK-180	0.1223	0.8777	0.3384	1.3795	0.6376	0.7104	0.0336	3.4343
mh11KK-187	0.1829	0.8171	0.3412	1.3876	0.6397	0.6574	0.2171	2.9071
mh11KK-191	0.1364	0.8636	0.3522	1.4198	0.6478	0.6998	0.5212	3.3140
mh12KK-043	0.2531	0.7469	0.2204	1.0763	0.5354	0.5792	0.4763	2.3699
mh12KK-045	0.4412	0.5588	0.0879	0.7730	0.3532	0.3626	0.8955	1.5672
mh12KK-046	0.1105	0.8895	0.4624	1.7958	0.7216	0.7480	0.5687	3.9449
mh12KK-092	0.3869	0.6131	0.1496	0.9142	0.4531	0.4689	0.6818	1.8796
mh12KK-093	0.5014	0.4986	0.0627	0.7123	0.2980	0.3345	0.0917	1.5011
mh12KK-202	0.1235	0.8765	0.5273	2.0847	0.7602	0.7338	0.5978	3.7356
mh13KK-047	0.2393	0.7607	0.1987	1.0256	0.5125	0.5685	0.0114	2.3113
mh13KK-213	0.1637	0.8363	0.2153	1.0643	0.5302	0.7550	0.0000	4.0395
mh13KK-217	0.0709	0.9291	0.4609	1.7899	0.7207	0.7910	0.0267	4.7474
mh13KK-218	0.0377	0.9623	0.7855	4.7692	0.8952	0.8656	0.2436	7.3473
mh13KK-223	0.0718	0.9282	0.5888	2.4327	0.7945	0.8019	0.6251	5.0081
mh13KK-225	0.1309	0.8691	0.4865	1.8955	0.7362	0.7202	0.4268	3.5560
mh13KK-226	0.3933	0.6067	0.1222	0.8521	0.4132	0.4373	0.5378	1.7741
mh14KK-048	0.2882	0.7118	0.1727	0.9664	0.4826	0.5456	0.0005	2.1951
mh14KK-068	0.2960	0.7040	0.2568	1.1636	0.5703	0.5543	0.3020	2.2380
mh14KK-101	0.5513	0.4487	0.0341	0.6374	0.2155	0.3058	0.0000	1.4390
mh15KK-066	0.1322	0.8678	0.3925	1.5443	0.6762	0.7086	0.1130	3.4141
mh15KK-067	0.2092	0.7908	0.3455	1.4000	0.6429	0.6402	0.3893	2.7695
mh15KK-095	0.2895	0.7105	0.1980	1.0240	0.5117	0.5532	0.2057	2.2329
mh15KK-104	0.5957	0.4043	0.0407	0.6554	0.2372	0.2461	0.2539	1.3256
mh16KK-049	0.1511	0.8489	0.4043	1.5833	0.6842	0.6973	0.0084	3.2878
mh16KK-096	0.3976	0.6024	0.1553	0.9270	0.4606	0.4599	0.5357	1.8483
mh16KK-255	0.1058	0.8942	0.5080	1.9919	0.7490	0.7486	0.0642	3.9543
mh16KK-302	0.0941	0.9059	0.5047	1.9766	0.7470	0.7620	0.1978	4.1750
mh17KK-052	0.1765	0.8235	0.2827	1.2287	0.5931	0.6593	0.1871	2.9227
mh17KK-053	0.2335	0.7665	0.2480	1.1422	0.5623	0.5969	0.1015	2.4736
mh17KK-054	0.2348	0.7652	0.1921	1.0106	0.5053	0.6325	0.0000	2.7085
mh17KK-055	0.3193	0.6807	0.4116	1.6083	0.6891	0.5581	0.0001	2.2555
mh17KK-077	0.6063	0.3937	0.0398	0.6531	0.2344	0.2422	0.6031	1.3187
mh17KK-105	0.9103	0.0897	0.0020	0.5247	0.0471	0.0460	1.0000	1.0482
mh17KK-110	0.5721	0.4279	0.0453	0.6675	0.2510	0.2634	0.3798	1.3567
mh17KK-272	0.1697	0.8303	0.2859	1.2371	0.5958	0.6559	0.0194	2.8943
mh18KK-285	0.1599	0.8401	0.3082	1.2959	0.6142	0.6601	0.3786	2.9305
mh18KK-293	0.1111	0.8889	0.4264	1.6600	0.6988	0.7387	0.0237	3.8057
mh19KK-056	0.3050	0.6950	0.1604	0.9385	0.4672	0.5301	0.1012	2.1231
mh19KK-057	0.3325	0.6675	0.1707	0.9618	0.4802	0.4890	0.7007	1.9534
mh19KK-299	0.1115	0.8885	0.3963	1.5570	0.6789	0.7245	0.0916	3.6110
mh19KK-301	0.5244	0.4756	0.0612	0.7083	0.2941	0.3054	0.4242	1.4384
mh20KK-058	0.1296	0.8704	0.4407	1.7123	0.7080	0.7232	0.8080	3.5940
mh20KK-059	0.2985	0.7015	0.2130	1.0588	0.5278	0.5203	0.9951	2.0801
mh20KK-307	0.1369	0.8631	0.2990	1.2713	0.6067	0.6991	0.0271	3.3076
mh21KK-315	0.0786	0.9214	0.5827	2.3942	0.7912	0.7865	0.6718	4.6484
mh21KK-316	0.1373	0.8627	0.4202	1.6382	0.6948	0.7072	0.6424	3.3985
mh21KK-320	0.0751	0.9249	0.5559	2.2358	0.7764	0.7914	0.4894	4.7555
mh21KK-324	0.1082	0.8918	0.4668	1.8134	0.7243	0.7404	0.4008	3.8302
mh22KK-060	0.1765	0.8235	0.2870	1.2400	0.5968	0.6649	0.1050	2.9726
mh22KK-061	0.1891	0.8109	0.3455	1.4000	0.6429	0.6462	0.2469	2.8158
mh22KK-064	0.5750	0.4250	0.0532	0.6882	0.2734	0.2642	0.8121	1.3581
mh22KK-069	0.2939	0.7061	0.2525	1.1532	0.5664	0.5446	0.5257	2.1905
mh22KK-303	0.3182	0.6818	0.1927	1.0120	0.5059	0.5013	0.6662	2.0011

MP: match probability; PD: power of discrimination; PE: power of exclusion; TPI: typical paternity index; H_o_: observed heterozygosity; H_e_: expected heterozygosity; p: p-value for Hardy–Weinberg equilibrium test; A_e_: effective number of alleles.

**Figure 5:**
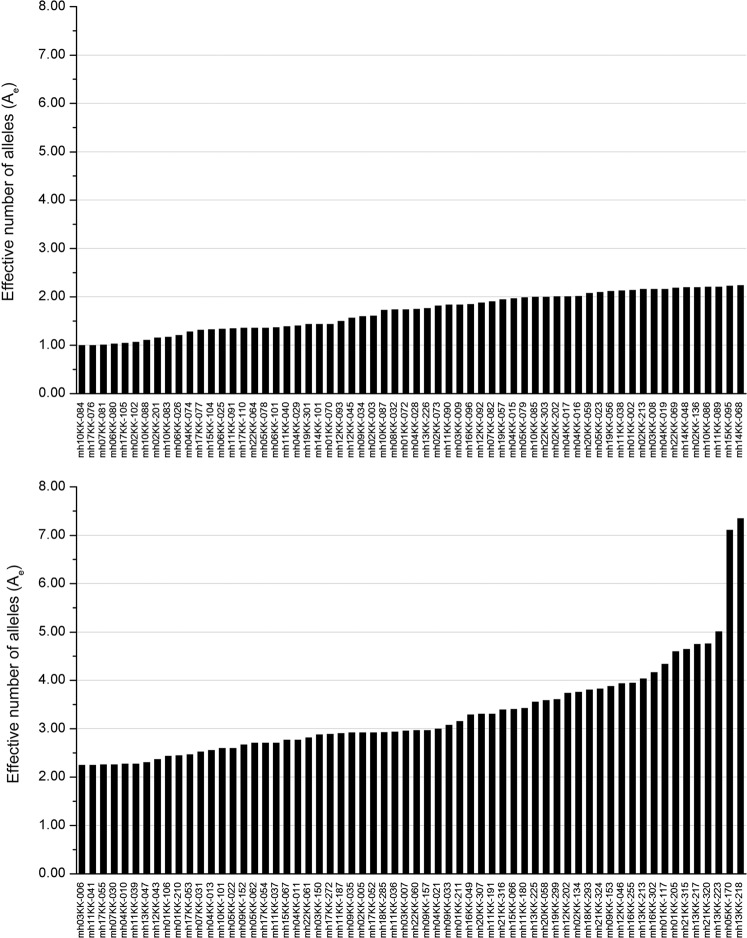
Histogram of the A_e_ values for the 124 microhaplotypes.

The PD values ranged from 0.0232 to 0.9623, with an average of 0.6799. The PD values for 90 loci were > 0.6, indicating that the individual identification capacity of the panel was high. The PEs for 66 loci were > 0.2, with 0.7855 (mh13KK-218) the highest PE value. Observed heterozygosity (H_o_) was 0.0070–0.8952, and expected heterozygosity (H_e_) was 0.0117–0.8656. The A_e_ values for 28 loci were > 3 (Fig. 5), and for another 23 loci, A_e_ was 2.5–3. Notably, the A_e_ values for mh13KK-218 and mh05KK-170 were even higher than 7.

To compare the individual identification capacities of the microhaplotypes and STRs, we summarized the PD and A_e_ values for the 20 microhaplotypes with the highest A_e_ values in the 124-plex panel and 20 commonly used forensic STRs (data under review in another manuscript) in Supplementary Table S3. The PD values for the microhaplotypes were 0.8691–0.9623 (0.9036 on average), which were very close to the PD range for STRs, 0.7794–0.9592 (0.9094 on average). The A_e_ values for the microhaplotypes and STRs were also similar. These data suggest that these 20 microhaplotypes are almost as effective as the commonly used forensic STRs for the identification of individuals.

To examine whether the microhaplotypes located on the same chromosome were linked to each other, we calculated LD. The *p*-values for pairwise linkage analyses are presented in Supplementary Table S4. Among the 124 microhaplotypes, 28 were linked in 10 pairs or groups (Supplementary Table S5) after correction for multiple testing (p < 0.0000065565). The locus with the highest A_e_ value within each linkage pair or group was used to calculate the combined forensic genetic parameters, whereas the other microhaplotypes within the linkage pairs or groups were not. Thus, based on 106 independent microhaplotypes, the combined match probability (CMP) and combined power of exclusion (CPE) were calculated to be 5.23 × 10^−66^ and (1–4.28 × 10^−16^), respectively.

## Discussion

Since the concept of microhaplotypes was introduced, their unique advantages as novel genetic markers in the field of forensics have been gradually demonstrated. Various research groups have conducted extensive research into microhaplotypes and provided data for different populations. Hiroaki *et al*. studied 27 multiple-SNP haplotype blocks in a Japanese population^4^. Chen and coworkers presented a novel panel of 26 microhaplotypes, with relatively high A_e_ (>3.0) and small sequence lengths (<50 bp)^17^. Voskoboinik *et al*. reported a panel of 10 highly polymorphic haplotypes, each containing more than 10 SNPs^10^. However, fewer surveys have been conducted with highly multiplexed systems. In this study, we developed a single-tube 124-plex assay for forensic microhaplotypes for use with next-generation sequencing.

The sequencing data from the 124-plex panel showed good intralocus and interlocus balance (Figs. 2 and 3), with over 90% of the reads classified as effective (Fig. 1). Mixture deconvolution is one of the major forensic applications for which microhaplotypes are advantageous, and it is noteworthy that the excellent intralocus balance characteristic of this panel provides a reliable foundation for mixture analyses.

Microhaplotypes are expected to provide a better solution than STRs to forensic mixture analyses because they circumvent the inference by stutters^3,18–20^. However, the extent to which microhaplotypes can improve mixture deconvolution has been unclear. Therefore, we undertook parallel mixture experiments and in-depth comparative analyses of CE-based STR and NGS-based microhaplotype genotyping. Our results show that only 38.10% and 4.76% of STR loci effectively analyzed 1:9 and 1:19 mixtures, respectively, whereas 99.19% and 91.94% of the microhaplotypes effectively analyzed the same mixtures, respectively (Table 2). The microhaplotypes were also superior to STRs in the analysis of forensic mixture because they avoided not only inference by stutters, but also the dropout of minor contributor alleles. It should be noted that these results were obtained by single experiments at each mixture ratio and needed further verification.

Probabilistic genotyping software, including LRmix^21^, STRmix^22^, and EuroForMix^23^, have been developed. Using semicontinuous or fully continuous models, these programs provide optional solutions for mixed STR profile deconvolution. As noted by Bennett *et al*.^24^, similar probabilistic calculations could also be helpful in mixed microhaplotype data analyses.

To evaluate their capacities to identify individuals and family/clan relationships in a Han Chinese population, we sequenced the DNA of 256 unrelated individuals. A statistical analysis showed that the majority of microhaplotypes sequenced were highly polymorphic and informative in the Gansu Han population. The CMPs for most commercial forensic STR kits range from 10^−17^ to 10^−26^ ^25–27^. In this study, the CMP for 106 microhaplotypes was 5.23 × 10^−66^, which is tens of orders of magnitude lower than those of STR multiplex systems. These data demonstrate that microhaplotypes are powerful genetic markers for the precise identification of individuals.

Some less polymorphic microhaplotypes in the Han Chinese population were kept in the 124-plex panel, including 2 markers which showed no genetic diversity. The ancestry inference capacity of these microhaplotypes has been extensively discussed by Kidd *et al*.^8,15,28,29^. Potential application of the 124-plex panel in ancestry inference awaits further studies.

### Conclusions

We have developed an NGS-based 124-plex panel of microhaplotypes. Mixture experiments showed that the microhaplotypes are superior to STRs in forensic mixture analysis because they avoid not only interference by stutters, but also the dropout of minor contributor alleles. The DNA of 256 Chinese Han individuals was sequenced with the 124-plex panel. The estimated forensic parameters showed that the 20 microhaplotype loci with the highest A_e_ values in the 124-plex panel were as efficient as STRs in the identification of individuals, and that CMP for 106 microhaplotypes was 5.23 × 10^−66^. These data demonstrate that the 124-plex microhaplotype panel provides an additional tool for forensic applications.

## Materials and Methods

### DNA samples

Blood samples were collected from unrelated Han Chinese individuals. Written informed consent was given by the blood donors and this work was approved by the Ethical Review Board of the Institute of Forensic Science, Ministry of Public Security of China (Beijing, China). All methods were performed in accordance with the relevant guidelines and regulations. DNA was extracted with the MagAttract M48 DNA Manual Kit (Qiagen, Limburg, Germany), according to the manufacturer’s guidelines. The extracted DNA samples were quantified with the Qubit dsDNA HS Assay Kit (Thermo Fisher Scientific, Waltham, MA, USA) on a Qubit fluorometer (Thermo Fisher Scientific).

The female genomic DNA standard 9947 A (Promega, Madison, WI, USA) was used in the sensitivity assays. Massive parallel sequencing was performed on a dilution series of genomic samples (1.0, 0.5, 0.2, or 0.1 ng). For the mixture experiments, standard genomic DNAs 9947 A and 2800 M (Promega) were mixed in ratios of 1:1, 1:3, 1:6, 1:9 and 1:19, to a total amount of 1.0 ng.

### Multiplex amplification

Primers were designed for the 130 microhaplotype loci reported by Kidd *et al*.^8^ with the Primer Premier 5.0 software^30^. After repeated optimization of the primer sequences and the PCR conditions, 124 microhaplotypes were successfully multiplexed in a single reaction system (Table 1). The PCRs were performed in a total volume of 20 μL containing 20 mM Tris-HCl (pH 8.3), 50 mM KCl, 1.6 mM MgCl_2_, 0.8 mg/ml bovine serum albumin, 0.2% (v/v) Tween 20, 3.2% (v/v) glycerol, 0.02% (w/v) NaN_3_, 200 mM each dNTP, 2 U of Taq DNA polymerase (Roche, Basel, Swiss), primer pairs (concentrations indicated in Table 1), and 1 ng of template DNA. The PCR conditions were 95 °C for 11 min, followed by 28 cycles of 30 s at 94 °C, 2 min at 60 °C, and 1 min at 72 °C, with a final elongation step at 60 °C for 60 min.

### Library preparation and sequencing

The PCR products were purified with the QIAquick 96 PCR Purification Kit (Qiagen) and the TruSeq DNA PCR-Free HT Kit (Illumina, San Diego, CA, USA) and used for library preparation, according to the manufacturer’s guidelines. The libraries were sequenced on a MiSeq FGx platform (Illumina) using the Miseq Reagent Kit v2 (Illumina), with a read length of 250 bases.

### Data analysis

FASTQ data were generated with the Miseq FGx Control Software 1.0.15.0 (Illumina). The MHTyper software^31^ was employed for microhaplotype allele calling, with the sequencing depth threshold set at 30 reads. The Hg19 human genome was used as the reference sequence. The allele frequencies and forensic statistical parameters (match probability, MP; power of discrimination, PD; power of exclusion, PE; typical paternity index, TPI) were calculated with Modified-PowerStat spreadsheet 1.2^32^. Arlequin 3.5^33^ was used to calculate the observed heterozygosity, expected heterozygosity, Hardy–Weinberg equilibrium, and linkage disequilibrium (LD). The effective number of alleles (A_e_) was calculated with the formula described in a previous publication^3^.

### CE-based STR genotyping

The GlobalFiler^®^ Kit (Thermo Fisher Scientific) was used for CE-based STR genotyping, according to the manufacturer’s recommendations. An aliquot of PCR product (1 µL) was added to 10 µL of deionized formamide (Thermo Fisher Scientific) containing the internal size standards. All samples were separated on a 3500XL Genetic Analyzer (Thermo Fisher Scientific) using POP™-4 Polymer (Thermo Fisher Scientific) and a 36 cm capillary array (Thermo Fisher Scientific). The GeneMapper^®^ ID-X software v4.0 (Thermo Fisher Scientific) was used for fragment sizing and allele calling.

## Supplementary information

Supplementary Figures S1-S19 and Supplementary Tables S1-S3 and S5.

Supplementary Table S4.
