# Supplementary material for: A 124-plex Microhaplotype Panel Based on Next-generation Sequencing Developed for Forensic Applications
Source: Sci Rep. 2020 Feb 6;10:1945. doi: 10.1038/s41598-020-58980-x (PMC7004988; doi:10.1038/s41598-020-58980-x)
Supplement: Supplementary file 1 — Supplementary Figures S1-S19 and Supplementary Tables S1-S3 and S5. [file 41598_2020_58980_MOESM1_ESM.pdf]

Supplementary Figures S1-S19 and Supplementary Tables S1-S3 and S5 for A 124-plex Microhaplotype Panel Based on Next-generation Sequencing Developed for Forensic Applications

Jing-Bo Pang[1,2,3,4], Min Rao[1,2,3,4], Qing-Feng Chen[1,2,4], An-Quan Ji[1,2,3], Chi Zhang[1,2], Ke-Lai Kang[1,2], Hao Wu[1,2], Jian Ye[1,2*], Sheng-Jie Nie[3*] & Le Wang[1,2,3*]

[1]National Engineering Laboratory for Forensic Science, Institute of Forensic Science, Ministry of Public Security, Beijing 100038, PR China. [2]Key Laboratory of Forensic Genetics of Ministry of Public Security, Institute of Forensic Science, Ministry of Public Security, Beijing 100038, PR China. [3]School of Forensic Medicine, Kunming Medical University, Kunming 650500, PR China. [4]These authors contributed equally to this work. [*]email: wangle_02@163.com; 879456764@qq.com; yejian77@126.com

Supplementary Fig. S1. Amplicon sizes of the 124-plex microhaplotype panel.

Supplementary Fig. S2. Histogram of the microhaplotype genotyping results obtained with 0.1 ng of 9947A.

Supplementary Fig. S3. Histogram of the microhaplotype genotyping results obtained with 0.2 ng of 9947A.

Supplementary Fig. S4. Histogram of the microhaplotype genotyping results obtained with 0.5 ng of 9947A.

Supplementary Fig. S5. Histogram of the microhaplotype genotyping results obtained with 1.0 ng of 9947A.

Supplementary Fig. S6. Histogram of the microhaplotype genotyping results obtained with 1.0 ng of 2800M.

Supplementary Fig. S7. STR genotyping profile obtained with 1.0 ng of 9947A.

Supplementary Fig. S8. STR genotyping profile obtained with 1.0 ng of 2800M.

Supplementary Fig. S9. STR genotyping profile for the 1:1 mixture.

Supplementary Fig. S10. STR genotyping profile for the 1:3 mixture.

Supplementary Fig. S11. STR genotyping profile for the 1:6 mixture.

Supplementary Fig. S12. STR genotyping profile for the 1:9 mixture.

Supplementary Fig. S13. STR genotyping profile for the 1:19 mixture.

Supplementary Fig. S14. Histogram of the microhaplotype genotyping results for the 1:1 mixture.

Supplementary Fig. S15. Histogram of the microhaplotype genotyping results for the 1:3 mixture.

Supplementary Fig. S16. Histogram of the microhaplotype genotyping results for the 1:6 mixture.

Supplementary Fig. S17. Histogram of the microhaplotype genotyping results for the 1:9 mixture.

Supplementary Fig. S18. Histogram of the microhaplotype genotyping results for the 1:19 mixture.

Supplementary Fig. S19. Drop-in artefacts, mh02KK003-GTC and mh20kk059-AG, in the 1:1 mixture.

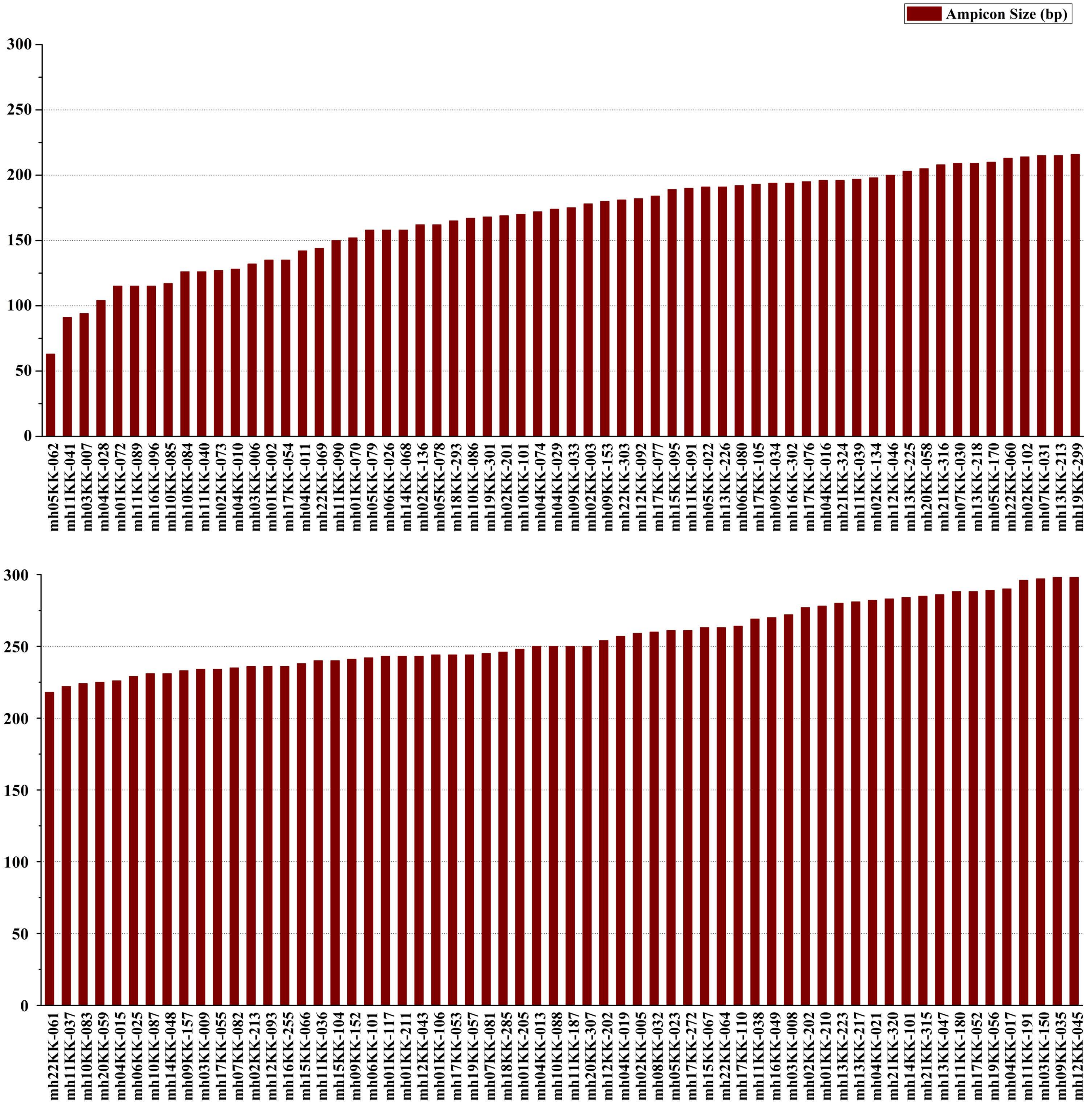

Ampicon Size (bp)
300
250
200
150
100
50
0
mh05KK-062
mh11KK-041
mh03KK-007
mh04KK-028
mh01KK-072
mh11KK-089
mh16KK-096
mh10KK-085
mh10KK-084
mh11KK-040
mh02KK-073
mh04KK-010
mh03KK-006
mh01KK-002
mh17KK-054
mh04KK-011
mh22KK-069
mh11KK-090
mh01KK-070
mh05KK-079
mh06KK-026
mh14KK-068
mh02KK-136
mh05KK-078
mh18KK-293
mh10KK-086
mh19KK-301
mh02KK-201
mh10KK-101
mh04KK-074
mh04KK-029
mh09KK-033
mh02KK-003
mh09KK-153
mh22KK-303
mh12KK-092
mh17KK-077
mh15KK-095
mh11KK-091
mh05KK-022
mh13KK-226
mh06KK-080
mh17KK-105
mh09KK-034
mh16KK-302
mh17KK-076
mh04KK-016
mh21KK-324
mh11KK-039
mh02KK-134
mh12KK-046
mh13KK-225
mh20KK-058
mh21KK-316
mh07KK-030
mh13KK-218
mh05KK-170
mh22KK-060
mh02KK-102
mh07KK-031
mh13KK-213
mh19KK-299
300
250
200
150
100
50
0
mh22KK-061
mh11KK-037
mh10KK-083
mh20KK-059
mh04KK-015
mh06KK-025
mh10KK-087
mh14KK-048
mh09KK-157
mh03KK-009
mh17KK-055
mh07KK-082
mh02KK-213
mh12KK-093
mh16KK-255
mh15KK-066
mh11KK-036
mh15KK-104
mh09KK-152
mh06KK-101
mh01KK-117
mh01KK-211
mh12KK-043
mh01KK-106
mh17KK-053
mh19KK-057
mh07KK-081
mh18KK-285
mh01KK-205
mh04KK-013
mh10KK-088
mh11KK-187
mh20KK-307
mh12KK-202
mh04KK-019
mh02KK-005
mh08KK-032
mh05KK-023
mh17KK-272
mh15KK-067
mh22KK-064
mh17KK-110
mh11KK-038
mh16KK-049
mh03KK-008
mh02KK-202
mh01KK-210
mh13KK-223
mh13KK-217
mh04KK-021
mh21KK-320
mh14KK-101
mh21KK-315
mh13KK-047
mh11KK-180
mh17KK-052
mh19KK-056
mh04KK-017
mh11KK-191
mh03KK-150
mh09KK-035
mh12KK-045

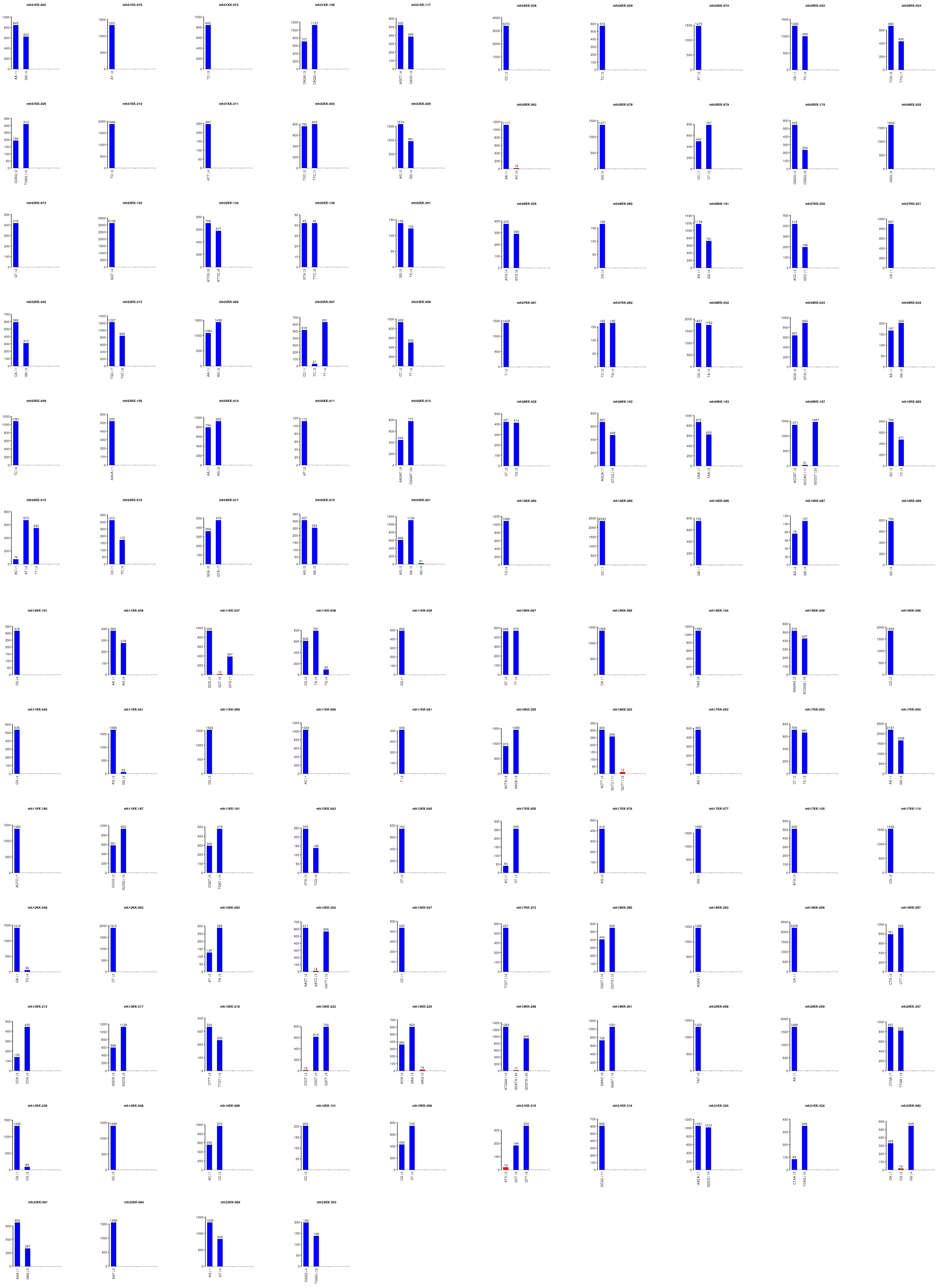

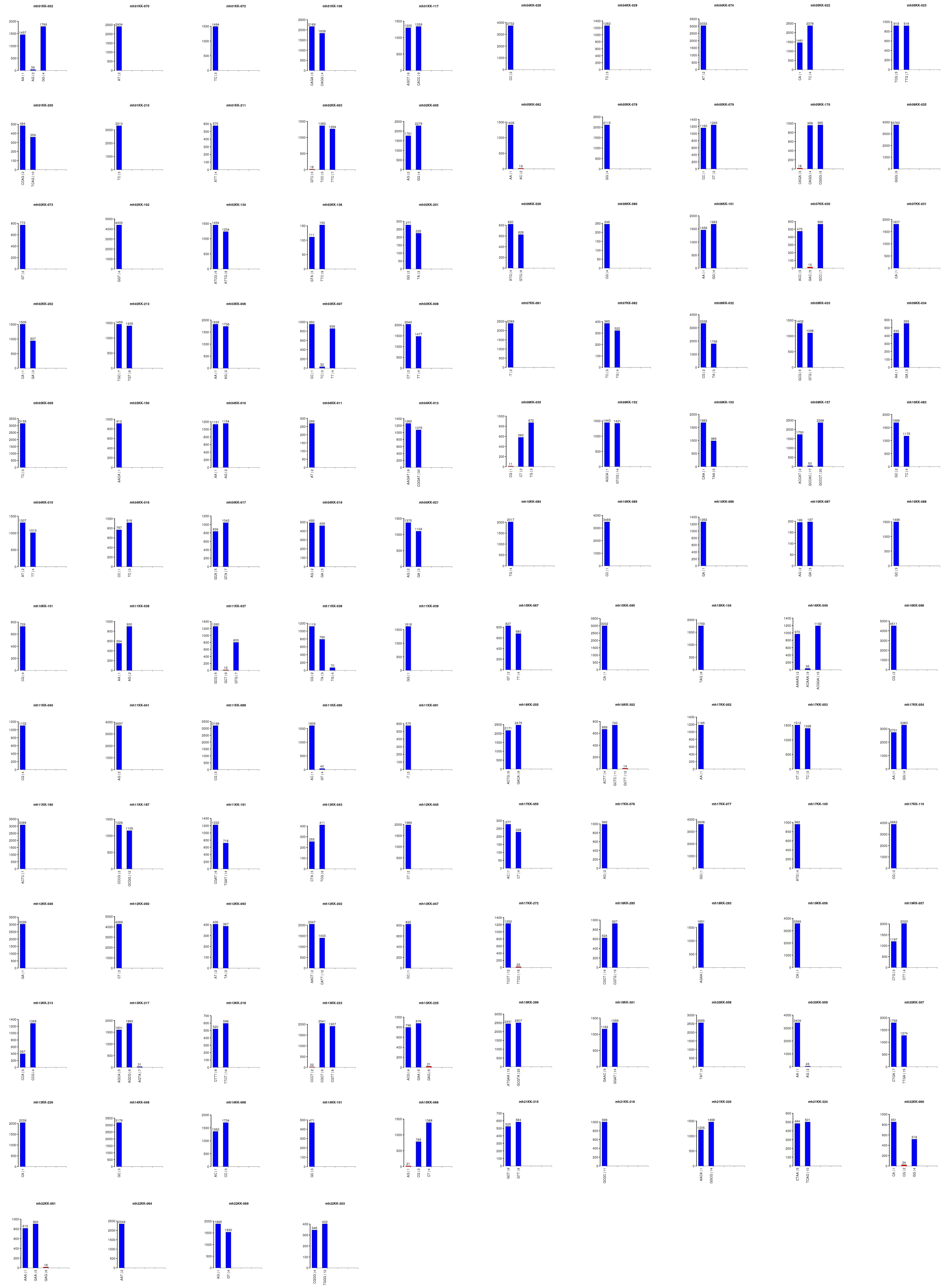

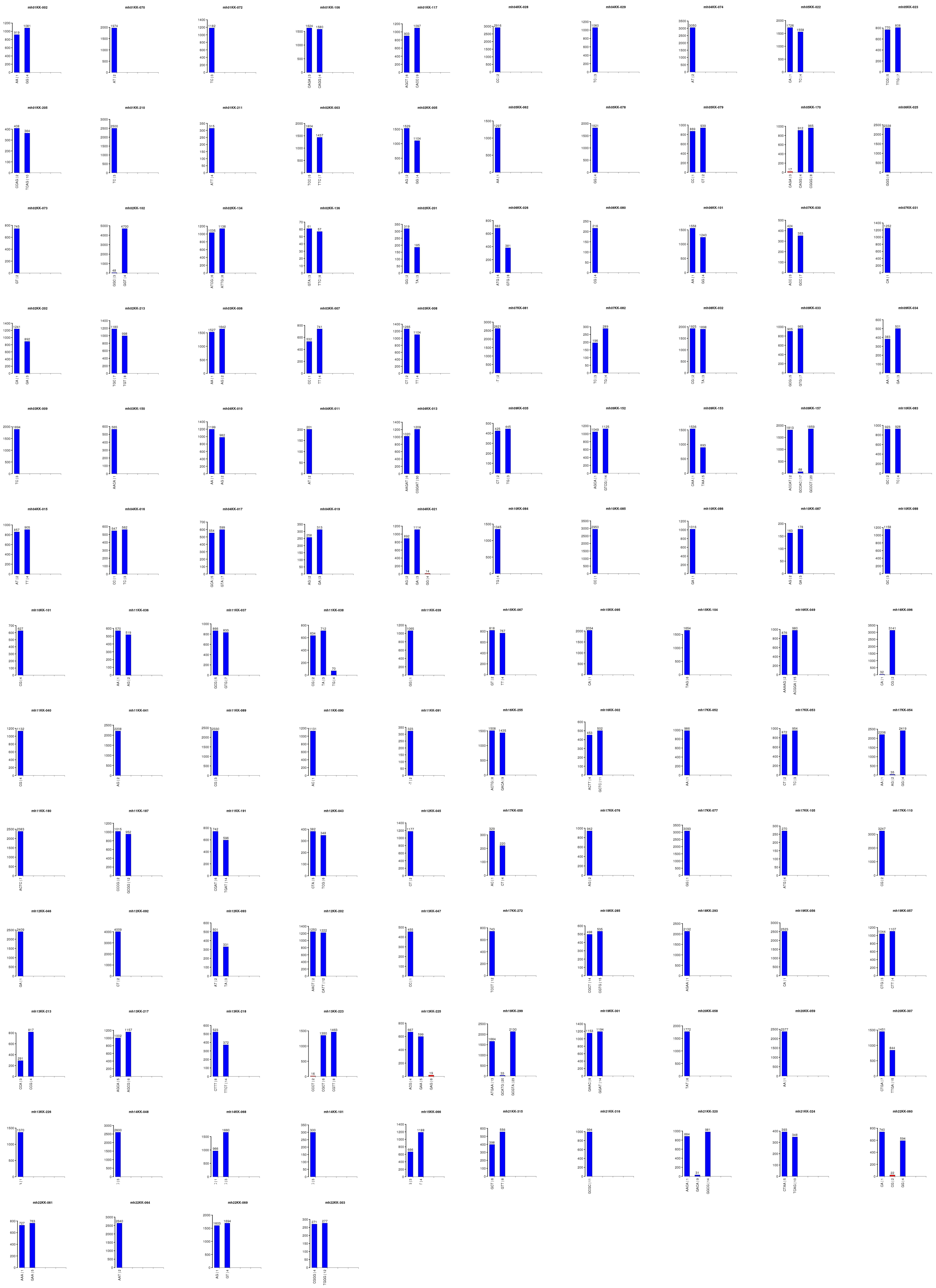

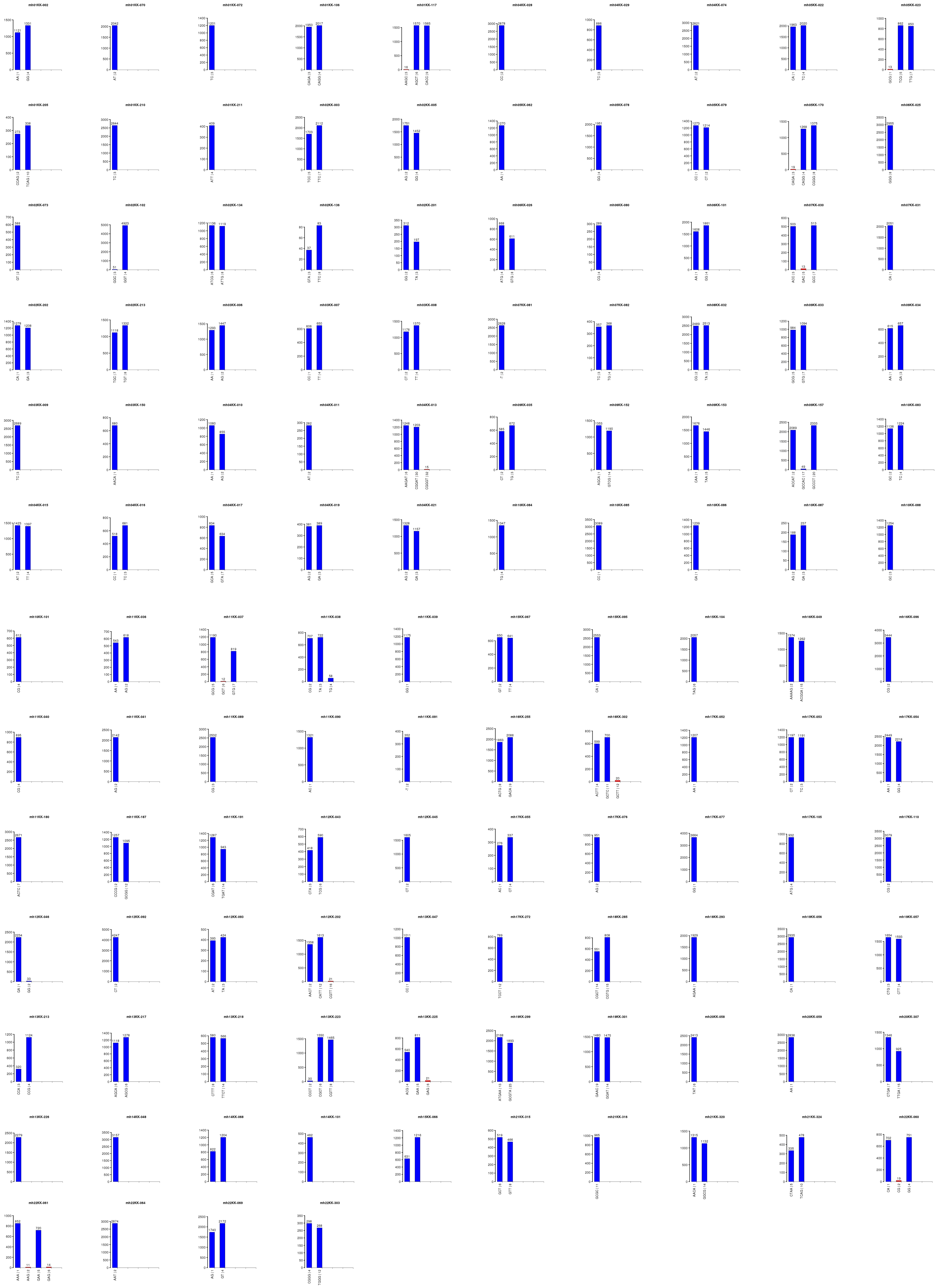

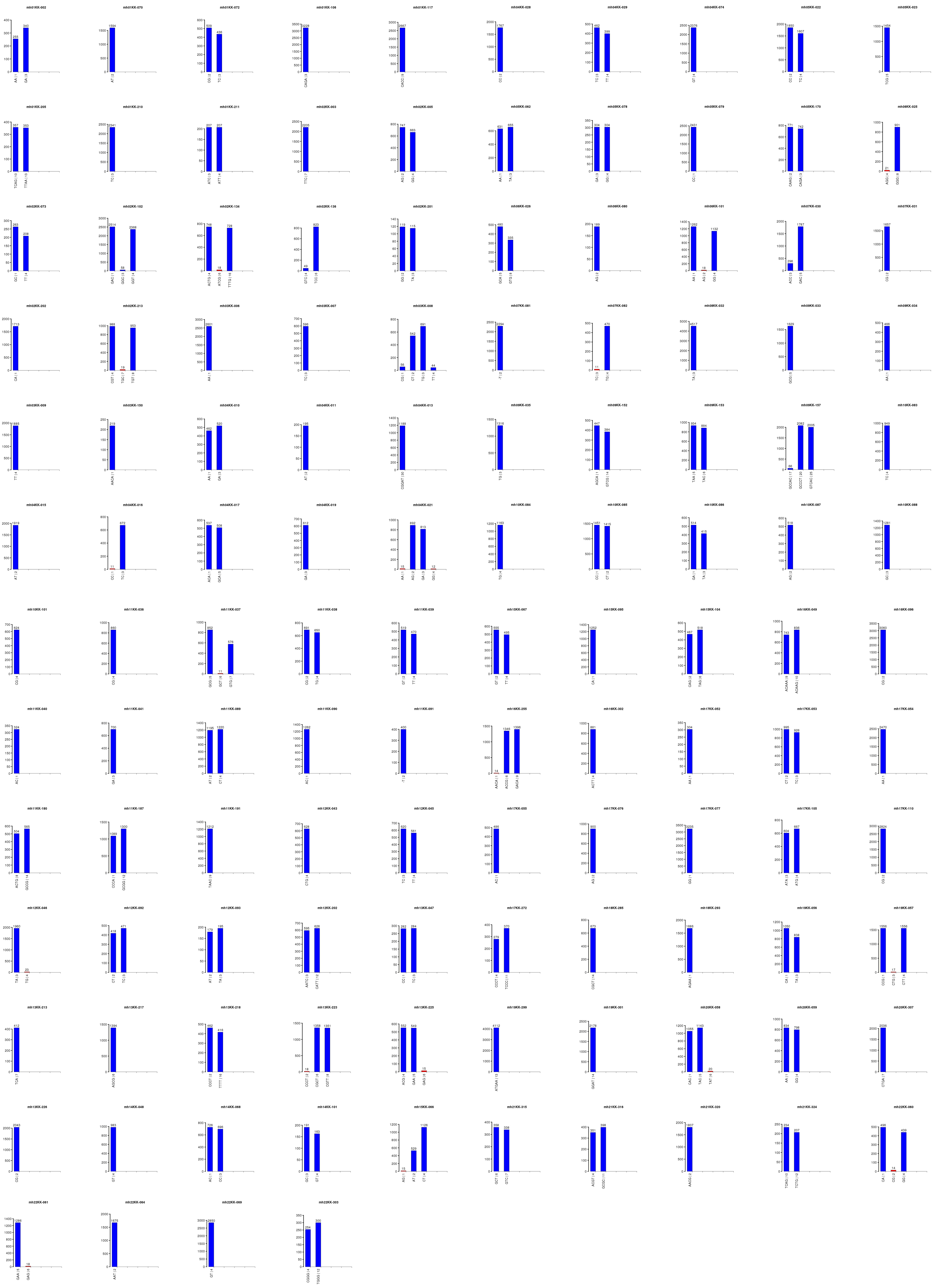

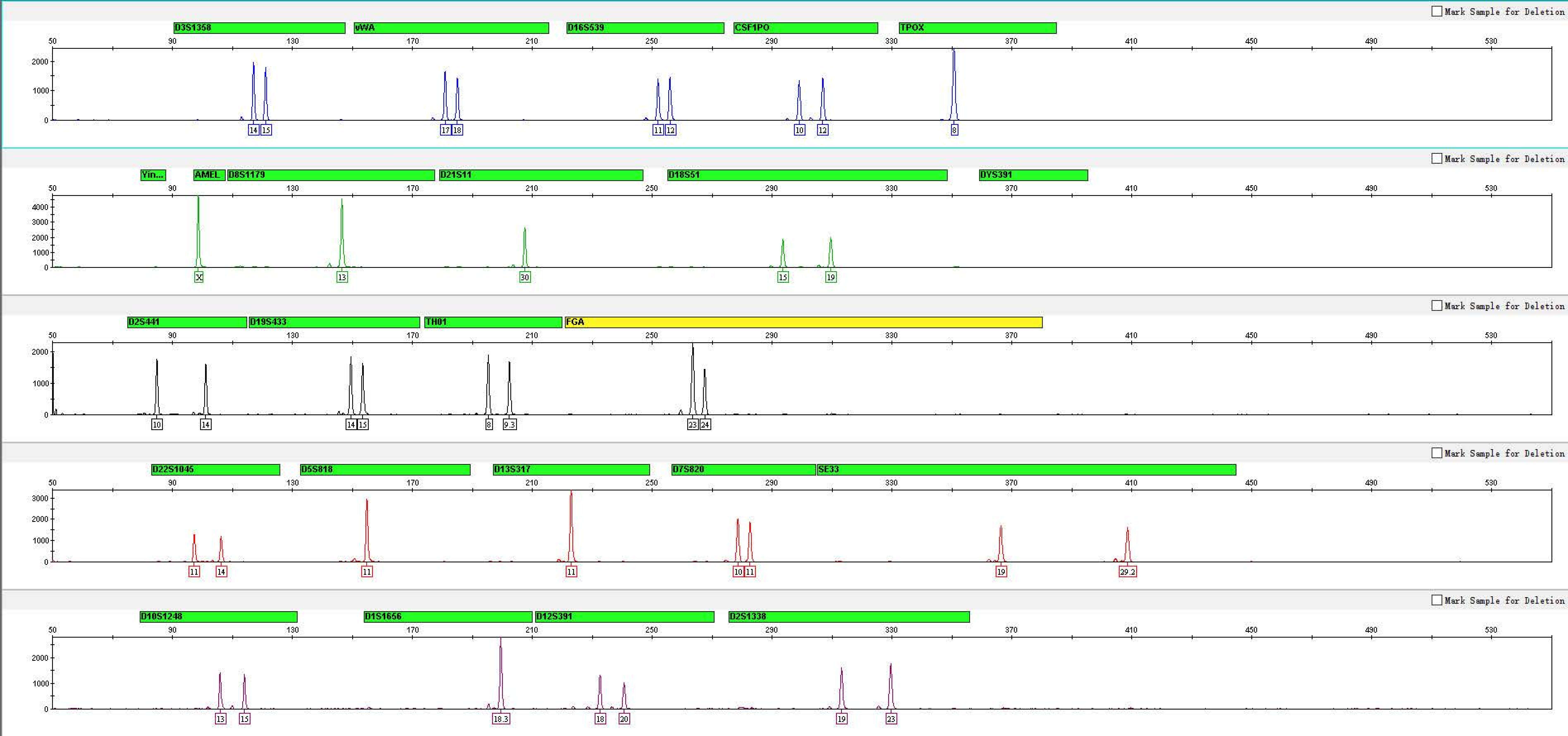
Mark Sample for Deletion
D3S1358
vWA
D16S539
CSF1PO
TPOX
14 15 17 18 11 12 10 12 8
Mark Sample for Deletion
Yin...
AMEL
D8S1179
D21S11
D18S51
DYS391
X 13 30 15 19
Mark Sample for Deletion
D2S441
D19S433
TH01
FGA
10 14 14 15 8 9.3 23 24
Mark Sample for Deletion
D22S1045
D5S818
D13S317
D7S820
SE33
11 14 11 11 10 11 19 29.2
Mark Sample for Deletion
D10S1248
D1S1656
D12S391
D2S1338
13 15 18.3 18 20 19 23

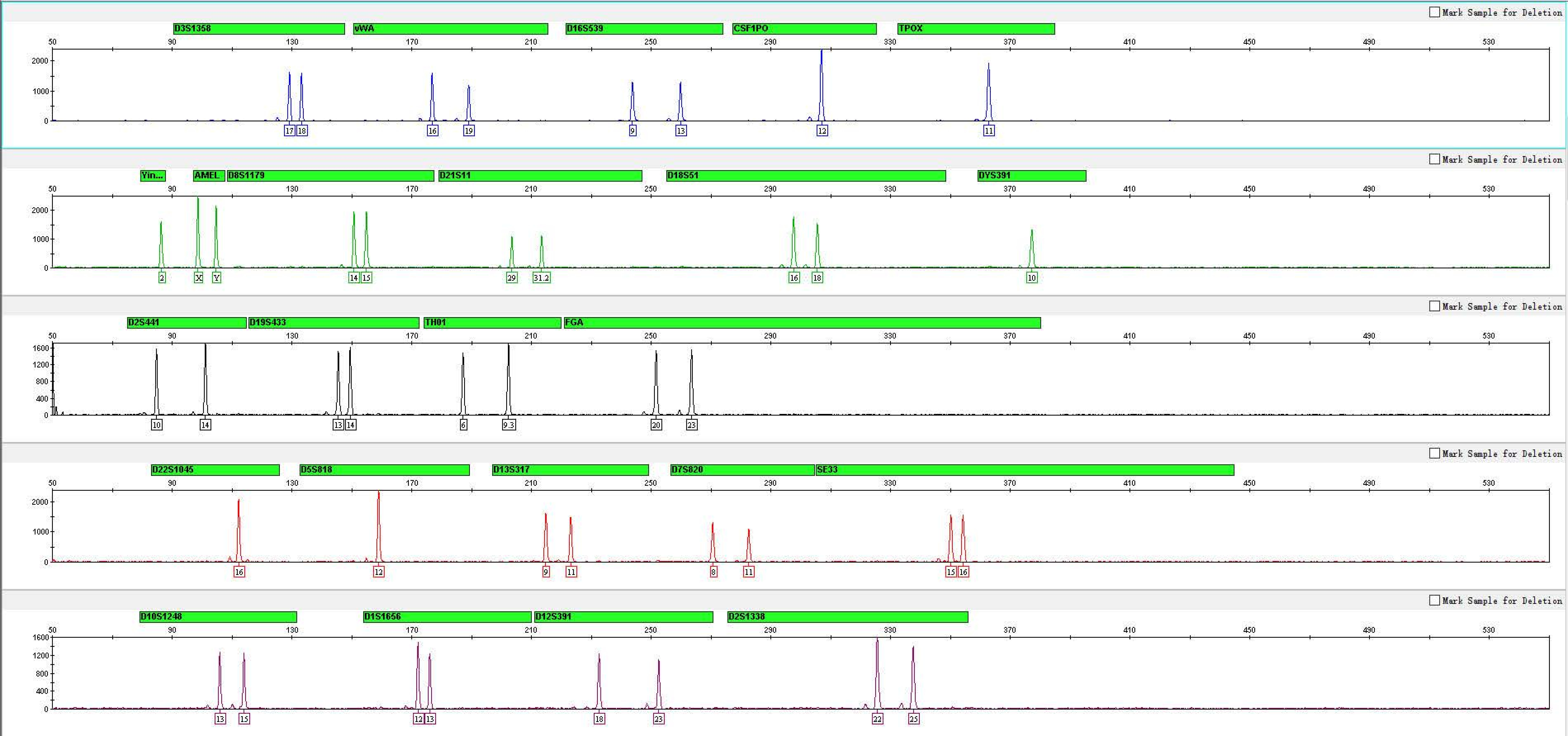
Mark Sample for Deletion
D3S1358
vWA
D16S539
CSF1PO
TPOX
17 18 16 19 9 13 12 11
Mark Sample for Deletion
Yin...
AMEL
D8S1179
D21S11
D18S51
DYS391
2 X Y 14 15 29 31.2 16 18 10
Mark Sample for Deletion
D2S441
D19S433
TH01
FGA
10 14 13 14 6 9.3 20 23
Mark Sample for Deletion
D22S1045
D5S818
D13S317
D7S820
SE33
16 12 9 11 8 11 15 16
Mark Sample for Deletion
D10S1248
D1S1656
D12S391
D2S1338
13 15 12 13 18 23 22 25

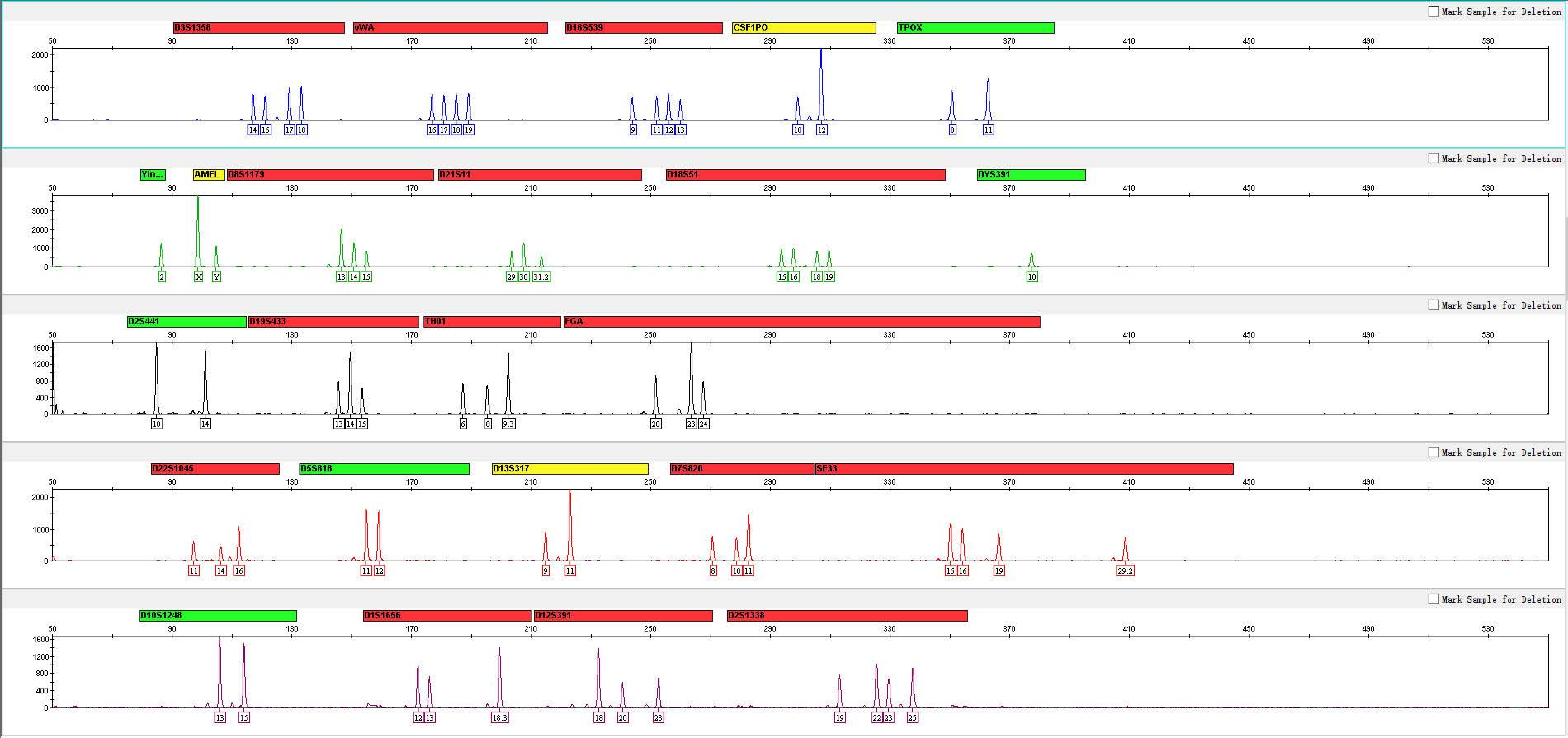

Mark Sample for Deletion
D3S1358
vWA
D16S539
CSF1PO
TPOX
14 15 17 18
16 17 18 19
9 11 12 13
10 12
8 11
Mark Sample for Deletion
Yin...
AMEL
D8S1179
D21S11
D18S51
DYS391
2
X Y
13 14 15
29 30 31.2
15 16 18 19
10
Mark Sample for Deletion
D2S441
D19S433
TH01
FGA
10 14
13 14 15
6 8 9.3
20 23 24
Mark Sample for Deletion
D22S1045
D5S818
D13S317
D7S820
SE33
11 14 16
11 12
9 11
8 10 11
15 16 19 29.2
Mark Sample for Deletion
D10S1248
D1S1656
D12S391
D2S1338
13 15
12 13 18.3
18 20 23
19 22 23 25

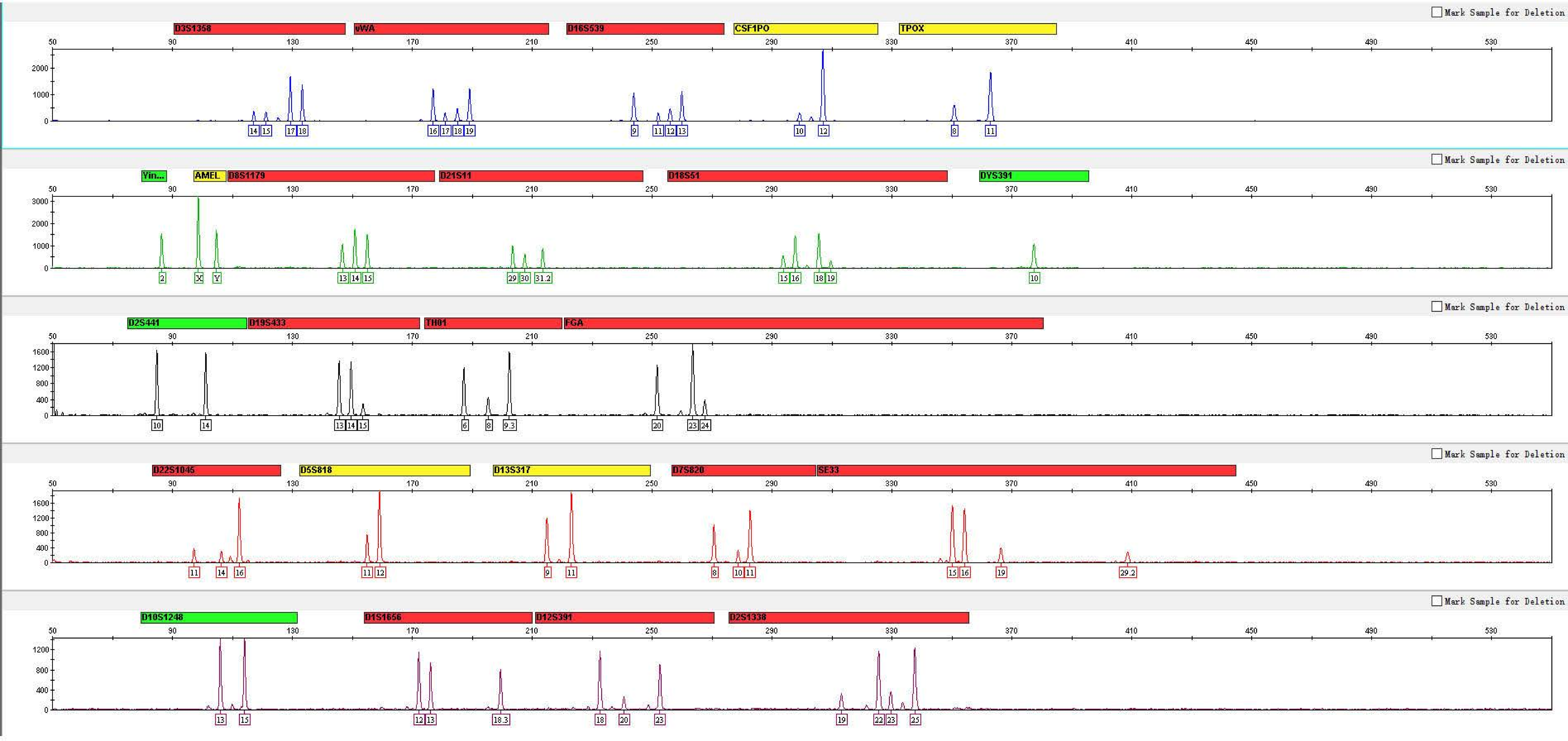
Mark Sample for Deletion
D3S1358
vWA
D16S539
CSF1PO
TPOX
14 15 17 18
16 17 18 19
9 11 12 13
10 12
8 11
Mark Sample for Deletion
Yin...
AMEL
D8S1179
D21S11
D18S51
DYS391
2
X Y
13 14 15
29 30 31.2
15 16 18 19
10
Mark Sample for Deletion
D2S441
D19S433
TH01
FGA
10 14
13 14 15
6 8 9.3
20 23 24
Mark Sample for Deletion
D22S1045
D5S818
D13S317
D7S820
SE33
11 14 16
11 12
9 11
8 10 11
15 16 19 29.2
Mark Sample for Deletion
D10S1248
D1S1656
D12S391
D2S1338
13 15
12 13 18.3
18 20 23
19 22 23 25

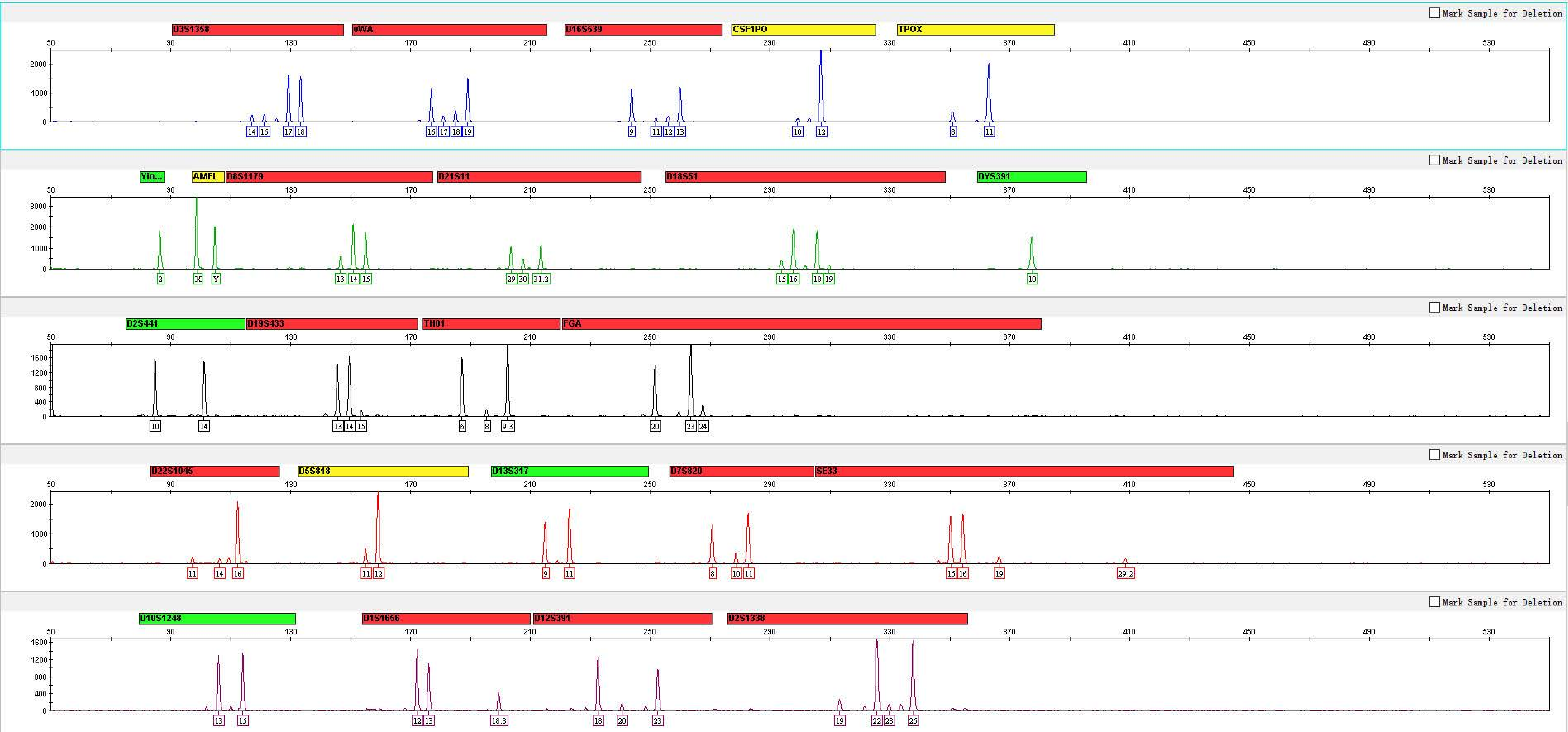
Mark Sample for Deletion
D3S1358
vWA
D16S539
CSF1PO
TPOX
14 15 17 18 16 17 18 19 9 11 12 13 10 12 8 11
Mark Sample for Deletion
Yin...
AMEL
D8S1179
D21S11
D18S51
DYS391
2 X Y 13 14 15 29 30 31.2 15 16 18 19 10
Mark Sample for Deletion
D2S441
D19S433
TH01
FGA
10 14 13 14 15 6 8 9.3 20 23 24
Mark Sample for Deletion
D22S1045
D5S818
D13S317
D7S820
SE33
11 14 16 11 12 9 11 8 10 11 15 16 19 29.2
Mark Sample for Deletion
D10S1248
D1S1656
D12S391
D2S1338
13 15 12 13 18.3 18 20 23 19 22 23 25

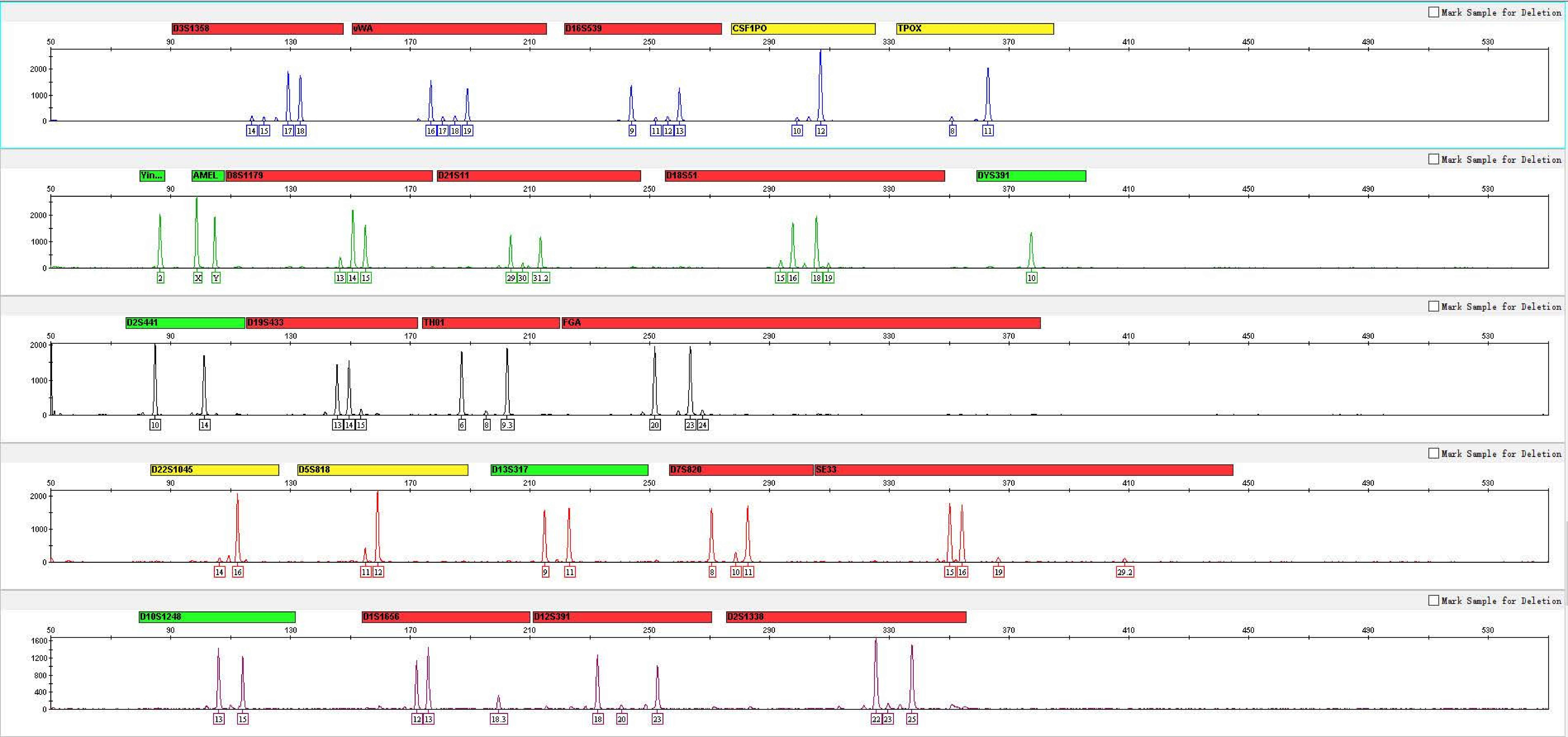
Mark Sample for Deletion
D3S1358
vWA
D16S539
CSF1PO
TPOX
14 15 17 18
16 17 18 19
9 11 12 13
10 12
8 11
Mark Sample for Deletion
Yin...
AMEL
D8S1179
D21S11
D18S51
DYS391
2
X Y
13 14 15
29 30 31.2
15 16 18 19
10
Mark Sample for Deletion
D2S441
D19S433
TH01
FGA
10 14
13 14 15
6 8 9.3
20 23 24
Mark Sample for Deletion
D22S1045
D5S818
D13S317
D7S820
SE33
14 16
11 12
9 11
8 10 11
15 16 19 29.2
Mark Sample for Deletion
D10S1248
D1S1656
D12S391
D2S1338
13 15
12 13 18.3
18 20 23
22 23 25

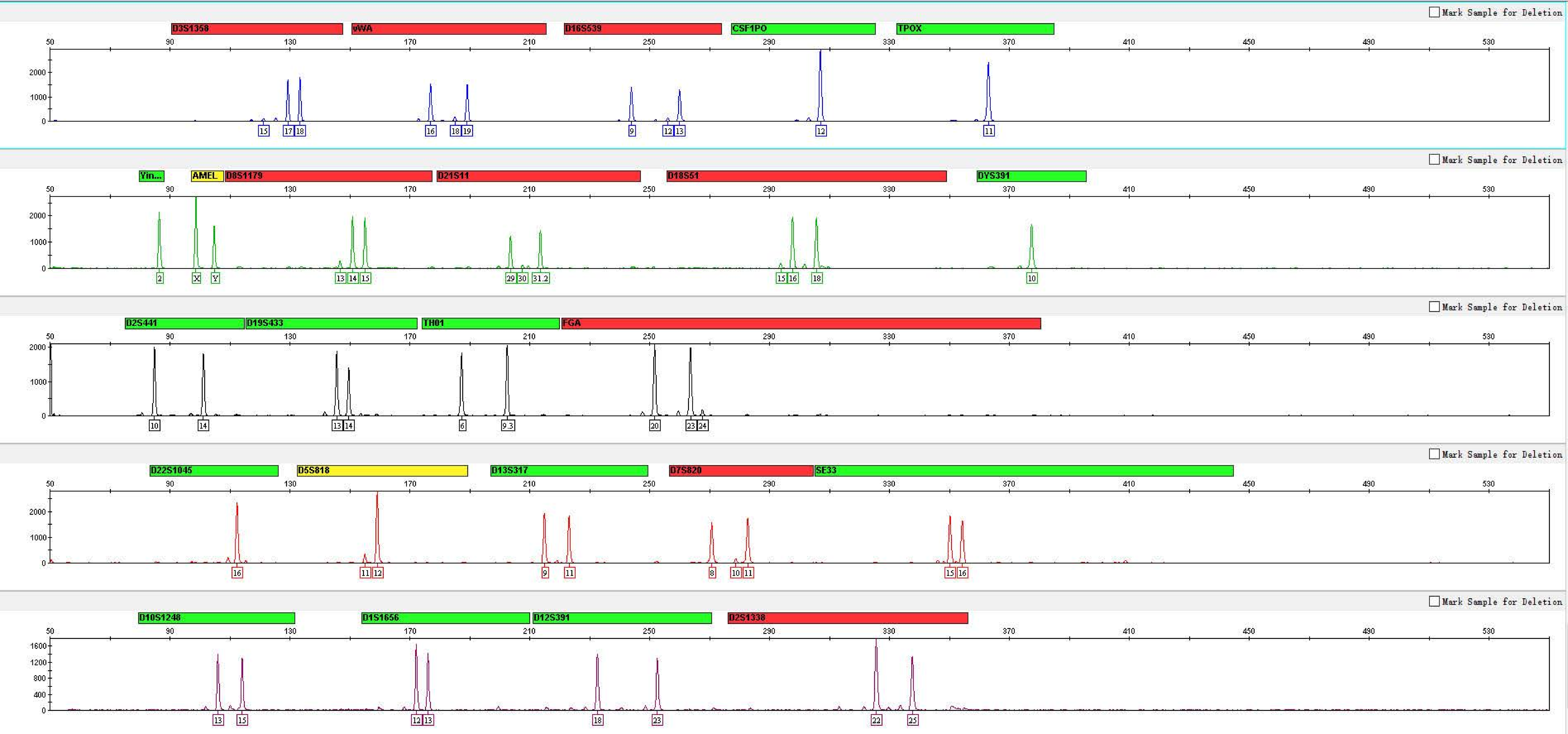

Mark Sample for Deletion
D3S1358
vWA
D16S539
CSF1PO
TPOX
50
90
130
170
210
250
290
330
370
410
450
490
530
2000
1000
0
15
17
18
16
18
19
9
12
13
12
11
Mark Sample for Deletion
Yin...
AMEL
D8S1179
D21S11
D18S51
DYS391
2
X
Y
13
14
15
29
30
31.2
15
16
18
10
Mark Sample for Deletion
D2S441
D19S433
TH01
FGA
10
14
13
14
6
9.3
20
23
24
Mark Sample for Deletion
D22S1045
D5S818
D13S317
D7S820
SE33
16
11
12
9
11
8
10
11
15
16
Mark Sample for Deletion
D10S1248
D1S1656
D12S391
D2S1338
1600
1200
800
400
0
13
15
12
13
18
23
22
25

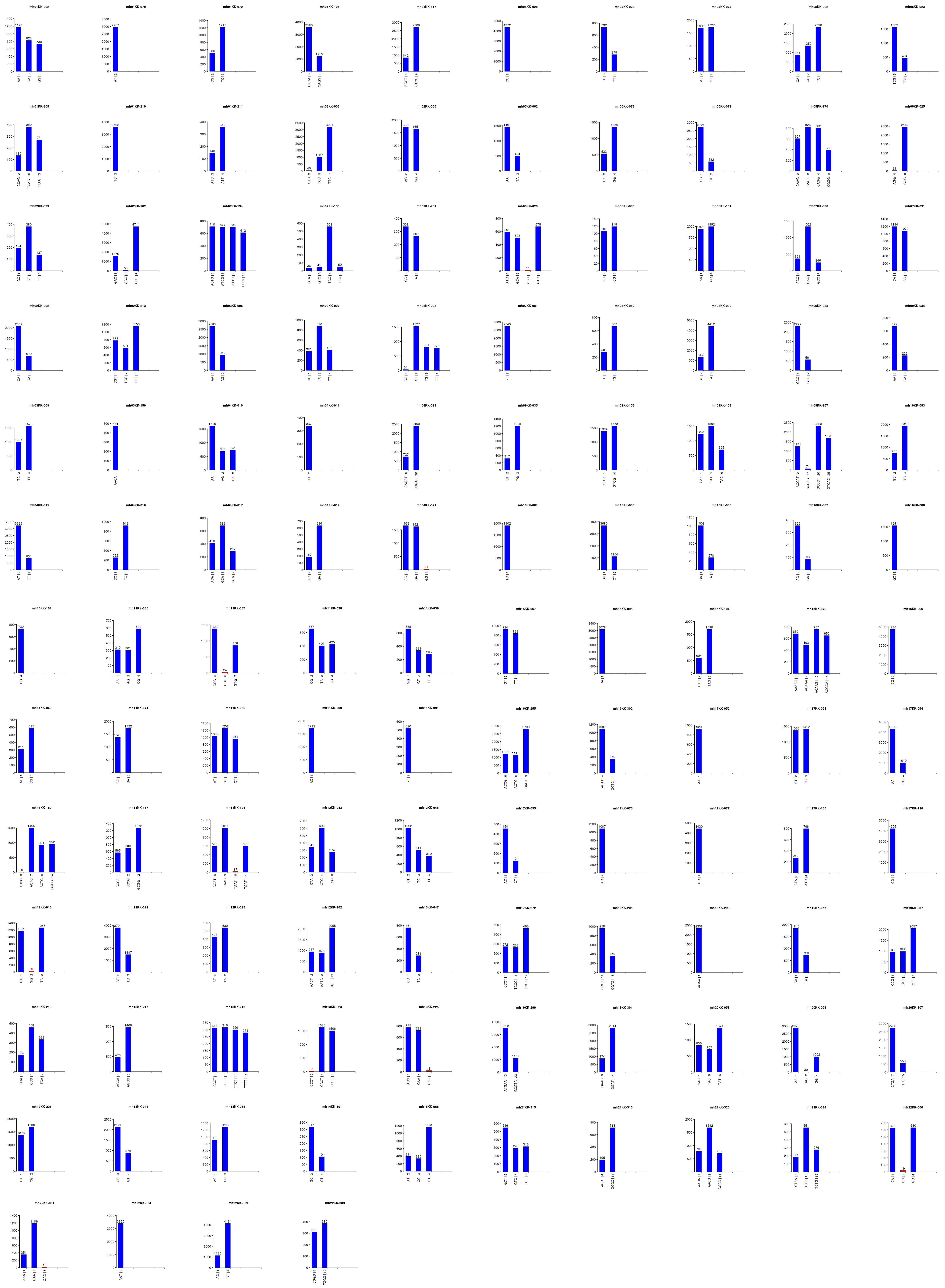

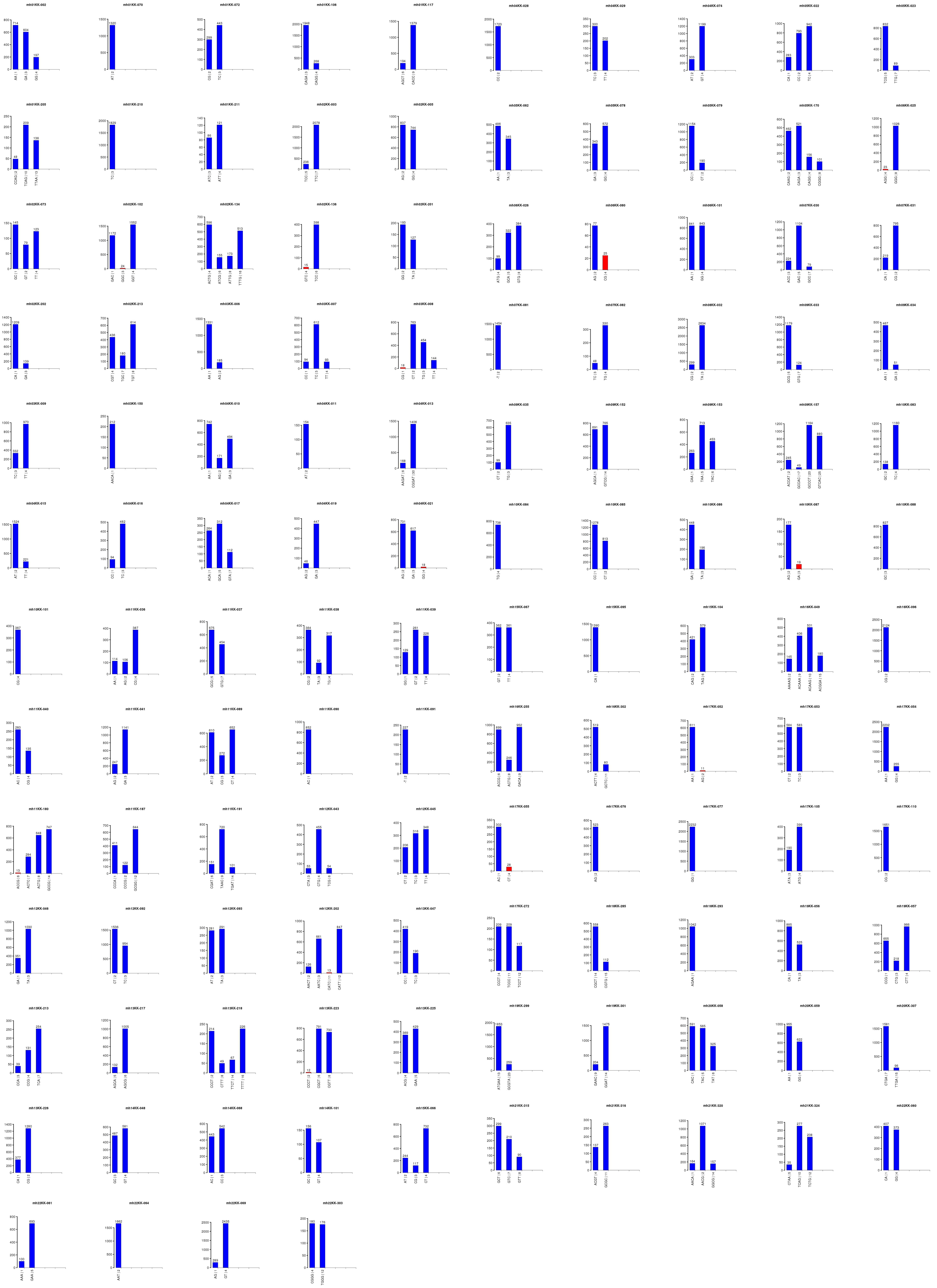

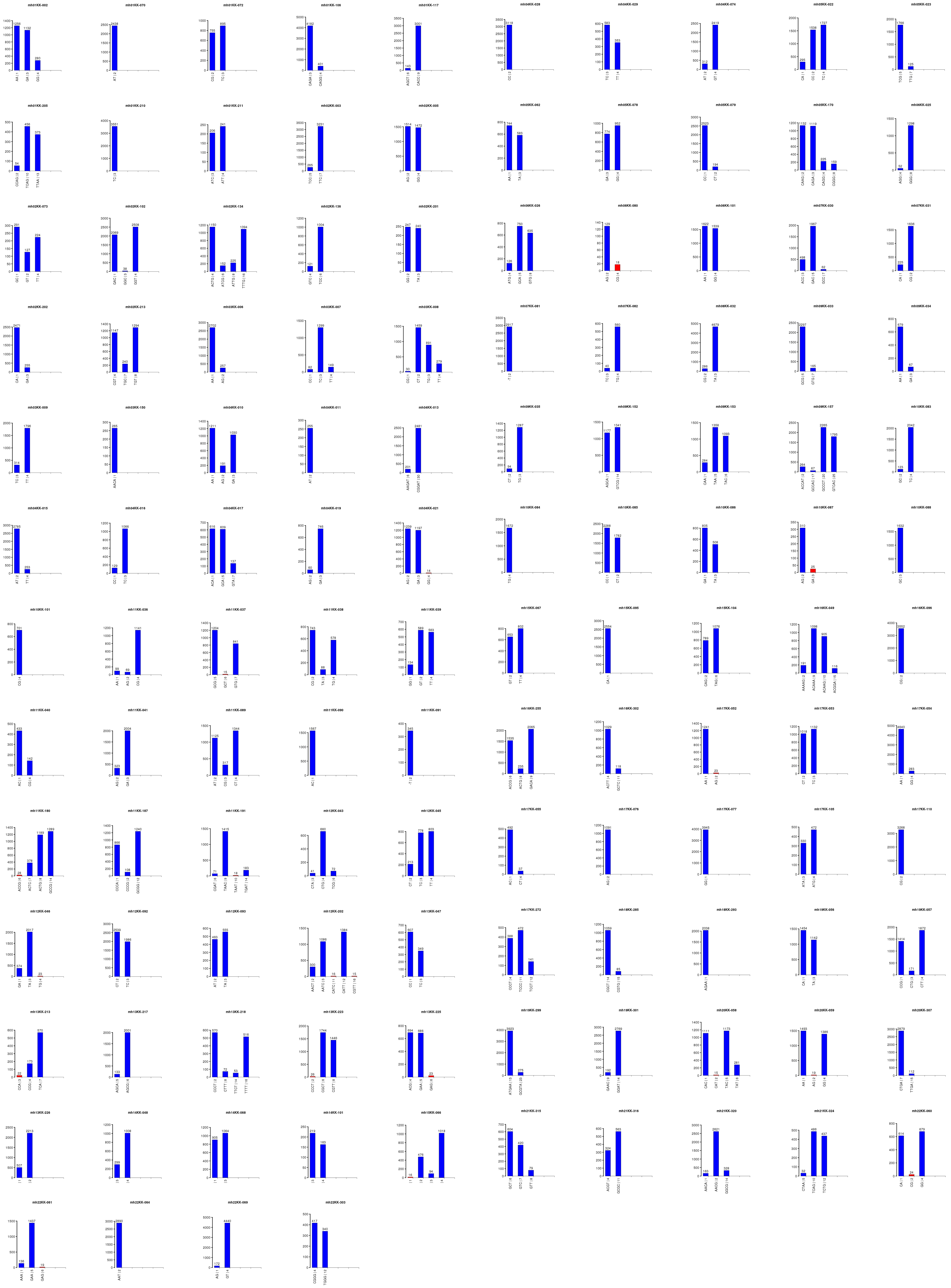

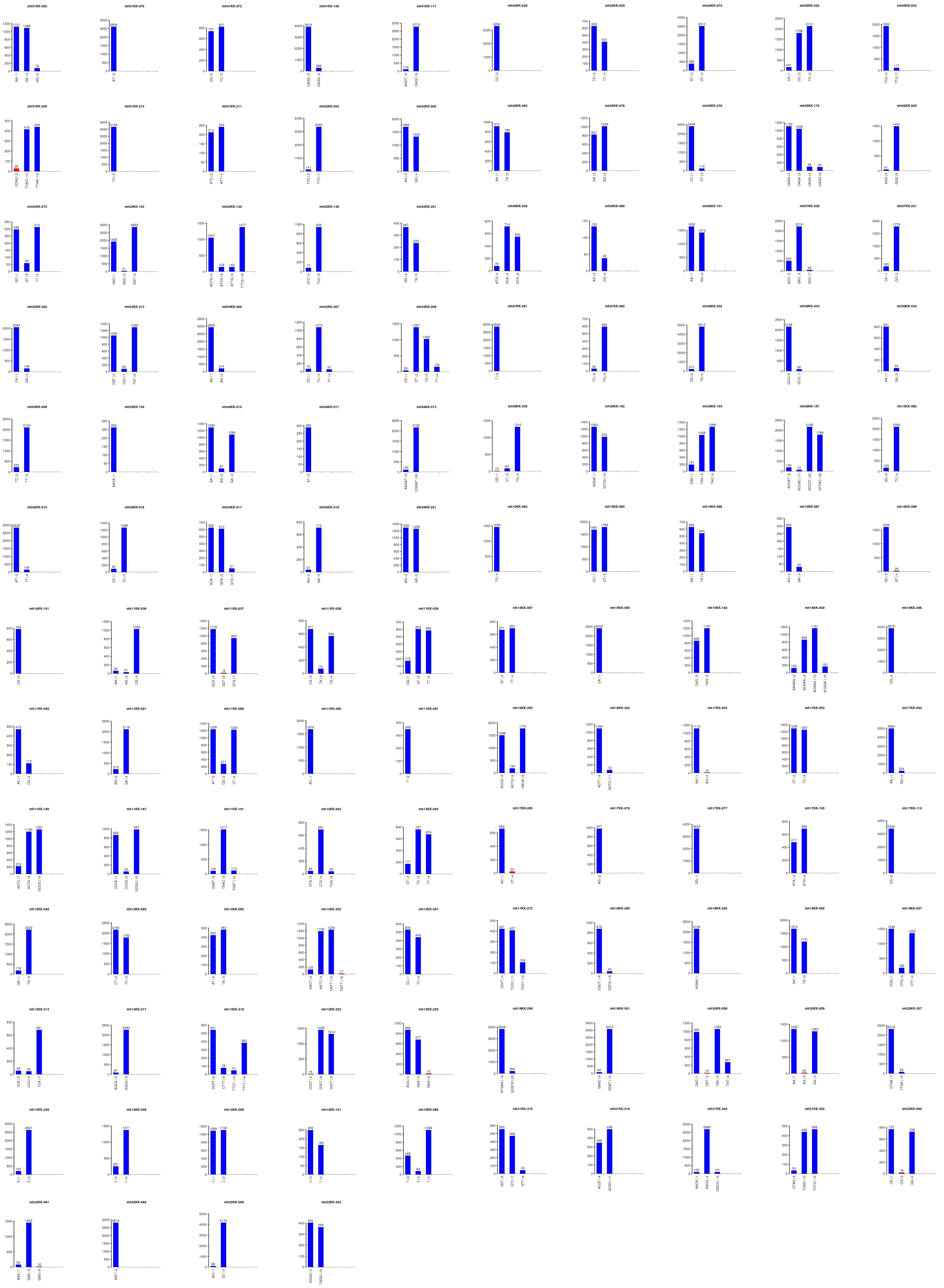

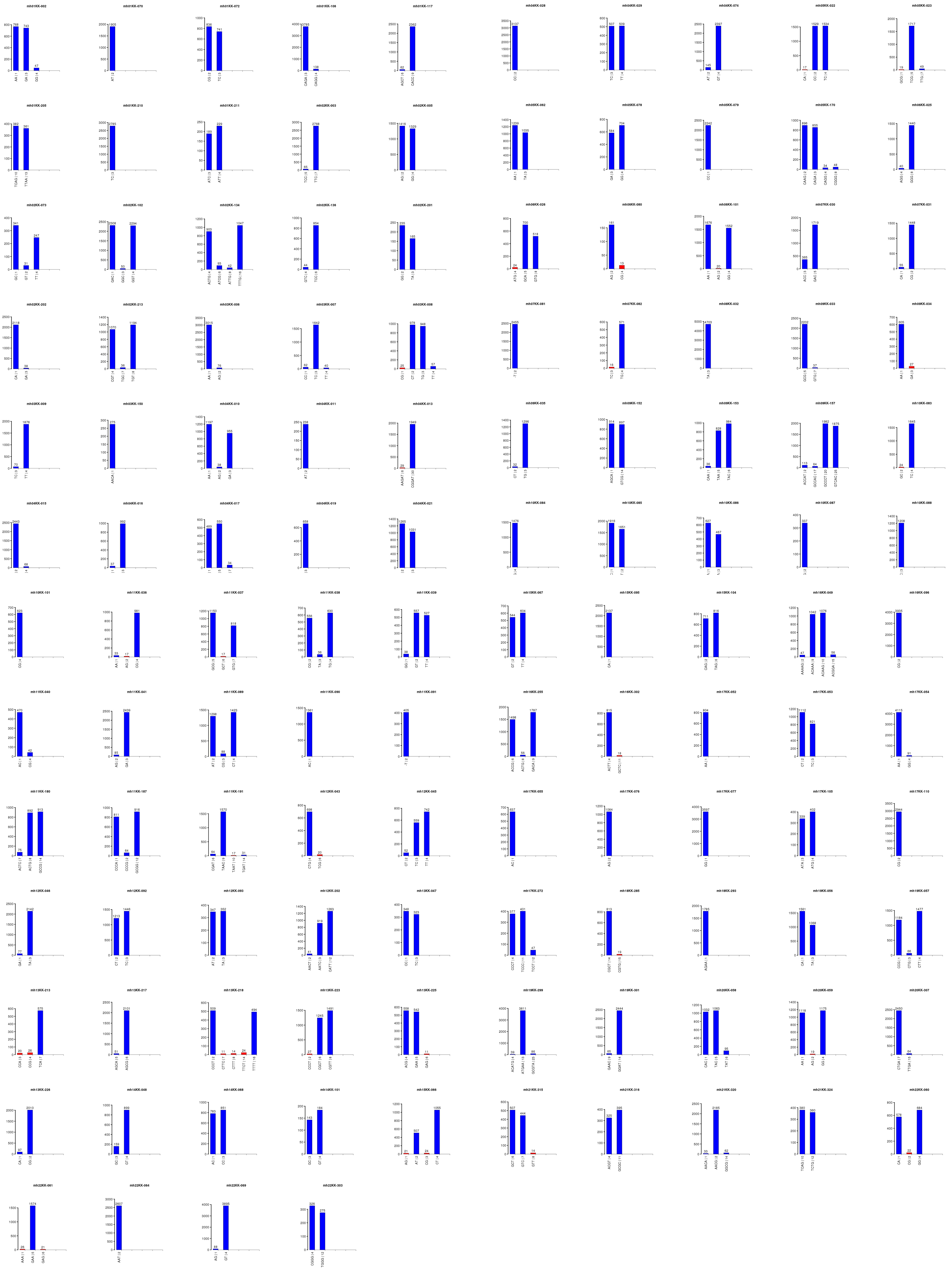

9947A

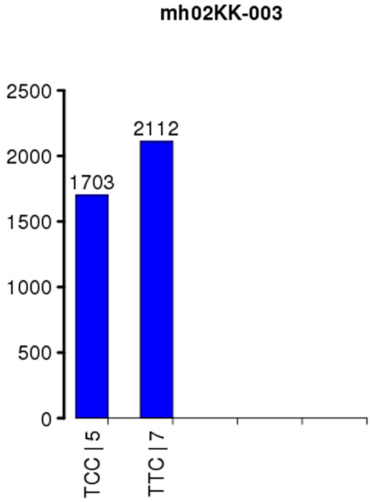

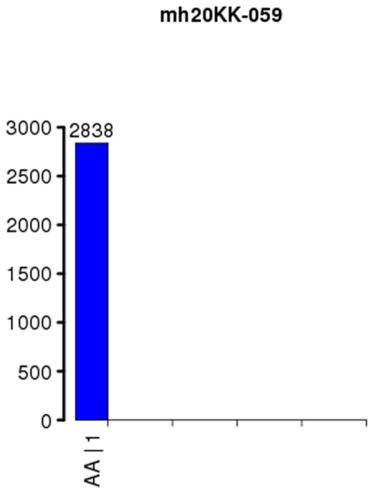

2800M

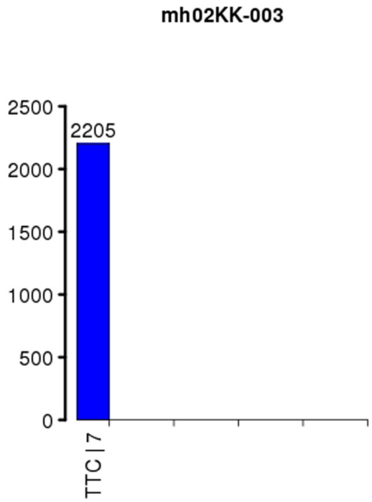

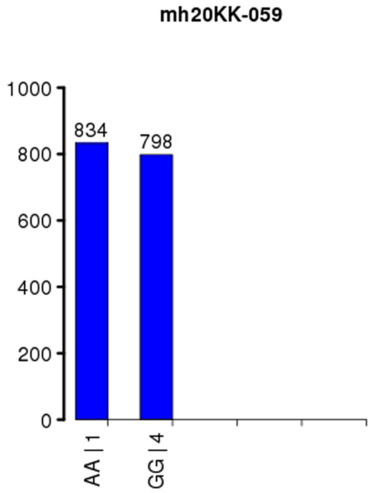

1:1

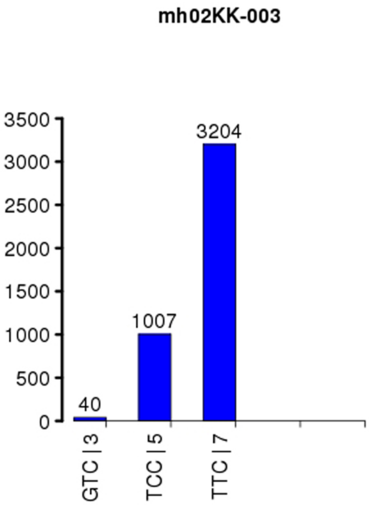

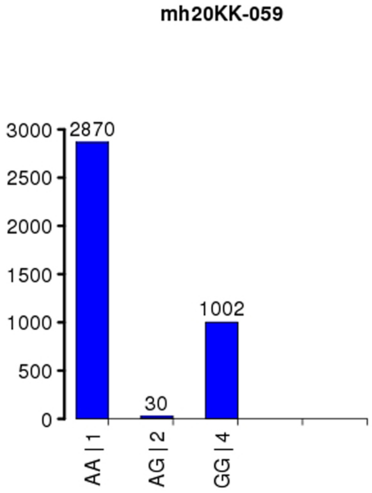

Supplementary Table S1. Microhaplotype loci covered in the 124-plex panel.

Microhaplotype	extent	No. of SNPs	SNPs
mh01KK-002	18	2	rs4528199/rs6604596
mh01KK-070	20	2	rs4846051/rs1801131
mh01KK-072	44	2	rs1251079/rs1251078
mh01KK-106	171	4	rs12123330/rs16840876/rs56212601/rs4468133
mh01KK-117	187	4	rs17413714/rs2772234/rs1610401/rs1610400
mh01KK-205	155	4	rs11810587/rs1336130/rs1533623/rs1533622
mh01KK-210	146	2	rs2165332/rs7536195
mh01KK-211	149	3	rs2490423/rs16835127/rs2341465
mh02KK-003	125	3	rs260694/rs11123719/rs11691107
mh02KK-005	157	2	rs2170607/rs10497052
mh02KK-073	64	2	rs1374748/rs7583554
mh02KK-102	137	3	rs2169812/rs6542783/rs2378217
mh02KK-134	104	4	rs12469721/rs3101043/rs3111398/rs72623112
mh02KK-136	71	3	rs6714835/rs6756898/rs12617010
mh02KK-201	115	2	rs1371048/rs786247
mh02KK-202	140	2	rs13422174/rs12464185
mh02KK-213	101	3	rs7568519/rs7577785/rs1519654
mh03KK-006	64	2	rs1919550/rs9873644
mh03KK-007	30	2	rs4513489/rs6441961
mh03KK-008	114	2	rs6808142/rs17030627
mh03KK-009	27	2	rs3732783/rs6280
mh03KK-150	185	4	rs1225051/rs1225050/rs1225049/rs1225048
mh04KK-010	35	2	rs3135123/rs495367
mh04KK-011	65	2	rs6855439/rs6531591
mh04KK-013	201	5	rs13131164/rs3775866/rs11725922/rs3775867/rs17088476
mh04KK-015	83	2	rs2584457/rs12648443
mh04KK-016	67	2	rs2851017/rs2032350
mh04KK-017	153	3	rs4699748/rs2584461/rs1442492
mh04KK-019	131	2	rs17731793/rs2122136
mh04KK-021	198	2	rs1280100/rs1280099
mh04KK-028	60	2	rs3762896/rs283413
mh04KK-029	23	2	rs59534319/rs971074
mh04KK-074	97	2	rs11932595/rs17085763
mh05KK-022	32	2	rs41461/rs41462
mh05KK-023	144	3	rs617938/rs2278325/rs2278324
mh05KK-062	19	2	rs870348/rs870347
mh05KK-078	60	2	rs2234234/rs2234233
mh05KK-079	91	2	rs2234232/rs41469
mh05KK-170	137	4	rs74865590/rs438055/rs370672/rs6555108
mh06KK-025	148	3	rs318453/rs9501899/rs11242810
mh06KK-026	75	3	rs4565296/rs4431439/rs179939
mh06KK-080	86	2	rs2056942/rs2056941
mh06KK-101	187	2	rs9356632/rs2180052
mh07KK-030	98	3	rs2330425/rs967066/rs10226425
mh07KK-031	86	2	rs17168174/rs10246622
mh07KK-081	82	2	rs41303343/rs28365094
mh07KK-082	95	2	rs150209521/rs713598
mh08KK-032	141	2	rs1390950/rs2898295
mh09KK-033	78	3	rs10815466/rs9408671/rs17431629
mh09KK-034	46	2	rs1408800/rs1408801
mh09KK-035	194	2	rs3118582/rs10776839
mh09KK-152	142	4	rs10867949/rs4282648/rs10780576/rs7046769
mh09KK-153	113	3	rs10125791/rs2987741/rs7047561
mh09KK-157	154	5	rs606141/rs8193001/rs56256724/rs2073578/rs633153

mh10KK-083	84	2	rs11568732/rs12248560
mh10KK-084	14	2	rs11572103/rs1058930
mh10KK-085	29	2	rs11572076/rs2275622
mh10KK-086	100	2	rs7909236/rs17110453
mh10KK-087	43	2	rs10884095/rs1452267
mh10KK-088	99	2	rs55897648/rs2515641
mh10KK-101	33	2	rs915907/rs915908
mh11KK-036	123	2	rs10500616/rs2499936
mh11KK-037	134	3	rs341065/rs10898849/rs395447
mh11KK-038	165	2	rs2303377/rs2303378
mh11KK-039	42	2	rs2288159/rs10891537
mh11KK-040	42	2	rs4938013/rs11214596
mh11KK-041	19	2	rs6277/rs6275
mh11KK-089	21	2	rs1124492/rs1124493
mh11KK-090	13	2	rs1079598/rs1079597
mh11KK-091	100	2	rs1799732/rs1799978
mh11KK-180	194	4	rs12802112/rs28631755/rs7112918/rs4752777
mh11KK-187	182	4	rs493442/rs17137917/rs551850/rs17137926
mh11KK-191	190	4	rs12421109/rs12289401/rs12420819/rs770566
mh12KK-043	111	3	rs11613749/rs11062734/rs17780102
mh12KK-045	186	2	rs2133298/rs3817446
mh12KK-046	72	2	rs1503767/rs11068953
mh12KK-092	84	2	rs2857234/rs2707209
mh12KK-093	56	2	rs11111391/rs7970874
mh12KK-202	154	4	rs10506052/rs4931233/rs10506053/rs4931234
mh13KK-047	166	2	rs806301/rs2066700
mh13KK-213	141	3	rs8181845/rs679482/rs9510616
mh13KK-217	193	4	rs7320507/rs9562648/rs9562649/rs2765614
mh13KK-218	146	4	rs1927847/rs9536429/rs7492234/rs9536430
mh13KK-223	154	4	rs1192204/rs1192205/rs3825483/rs3825481
mh13KK-225	97	3	rs4884651/rs9529023/rs7329287
mh13KK-226	118	2	rs721367/rs2892698
mh14KK-048	159	2	rs12717560/rs12878166
mh14KK-068	21	2	rs1887063/rs1887064
mh14KK-101	96	2	rs28529526/rs10134526
mh15KK-066	75	2	rs1063902/rs4219
mh15KK-067	122	2	rs701463/rs701464
mh15KK-095	100	2	rs2433354/rs2459391
mh15KK-104	138	3	rs11631544/rs10152453/rs80047978
mh16KK-049	174	5	rs9937467/rs17670098/rs17670111/rs12929083/rs9926495
mh16KK-096	38	2	rs1805007/rs885479
mh16KK-255	143	4	rs16956011/rs3934955/rs3934956/rs4073828
mh16KK-302	114	4	rs1395579/rs1395580/rs1395582/rs9939248
mh17KK-052	187	2	rs1059504/rs8327
mh17KK-053	83	2	rs3760370/rs3760371
mh17KK-054	43	2	rs2233362/rs634370
mh17KK-055	60	2	rs11868709/rs9907137
mh17KK-076	41	2	rs241027/rs528912185
mh17KK-077	73	2	rs4074461/rs4074462
mh17KK-105	130	3	rs1052553/rs17652121/rs11568305
mh17KK-110	192	2	rs9908046/rs8075367
mh17KK-272	131	4	rs2934897/rs7207239/rs16955257/rs7212184
mh18KK-285	136	4	rs16940823/rs17187688/rs17187695/rs1945150
mh18KK-293	83	4	rs621320/rs621340/rs678179/rs621766
mh19KK-056	201	2	rs1055919/rs2271057
mh19KK-057	115	3	rs17717333/rs12462026/rs7250849
mh19KK-299	154	5	rs12985452/rs4932999/rs4932769/rs2361019/rs2860462

mh19KK-301	64	4	rs10408594/rs11084040/rs10408037/rs8104441
mh20KK-058	106	3	rs6122890/rs6095836/rs6012881
mh20KK-059	97	2	rs10854214/rs10854215
mh20KK-307	141	4	rs6044080/rs17674942/rs6044081/rs16997830
mh21KK-315	146	3	rs8126597/rs8131148/rs6517971
mh21KK-316	135	4	rs961302/rs17002090/rs961301/rs2830208
mh21KK-320	186	4	rs2838081/rs2838082/rs78902658/rs2838083
mh21KK-324	159	4	rs6518223/rs2838868/rs7279250/rs8133697
mh22KK-060	65	2	rs4818/rs4680
mh22KK-061	147	3	rs763040/rs5764924/rs763041
mh22KK-064	210	4	rs136177/rs73885319/rs60910145/rs71785313
mh22KK-069	79	2	rs8137373/rs2235845
mh22KK-303	95	4	rs4633/rs6267/rs740602/rs76452330

Supplementary Table S2. Sensitivity of the 124-plex microhaplotype panel.

Input DNA	Number of genotyped loci	Percentage of genotyped loci
1.0 ng	124	100%
0.5 ng	124	100%
0.2 ng	124	100%
0.1 ng	124	100%

Supplementary Table S3. Comparison of PD and A_e values of microhaplotypes and STRs.

Microhaplotype	PD	A_e	STR	PD	A_e
mh13KK-218	0.9623	7.3473	FGA	0.9592	6.9794
mh05KK-170	0.9620	7.1065	D8S1179	0.9569	6.4206
mh13KK-223	0.9282	5.0081	D2S1338	0.9568	6.7552
mh21KK-320	0.9249	4.7555	D12S391	0.9490	6.0847
mh13KK-217	0.9291	4.7474	D18S51	0.9433	5.6526
mh21KK-315	0.9214	4.6484	D19S433	0.9376	5.3119
mh01KK-205	0.9174	4.5984	D1S1656	0.9368	5.0940
mh01KK-117	0.9142	4.3362	D21S11	0.9361	5.2887
mh16KK-302	0.9059	4.1750	VWA	0.9330	5.1018
mh13KK-213	0.8363	4.0395	D13S317	0.9276	4.9167
mh16KK-255	0.8942	3.9543	D16S539	0.9229	4.7438
mh12KK-046	0.8895	3.9449	D5S818	0.9103	4.4115
mh09KK-153	0.8969	3.8810	D7S820	0.9095	4.3337
mh21KK-324	0.8918	3.8302	D1S1679	0.8950	4.9042
mh18KK-293	0.8889	3.8057	CSF1PO	0.8823	3.7802
mh02KK-134	0.9048	3.7641	D6S474	0.8810	3.7789
mh12KK-202	0.8765	3.7356	D17S974	0.8686	3.4058
mh19KK-299	0.8885	3.6110	D3S1358	0.8659	3.5700
mh20KK-058	0.8704	3.5940	TH01	0.8362	3.0091
mh13KK-225	0.8691	3.5560	TPOX	0.7794	2.4852
Average	0.9036	4.4220	Average	0.9094	4.8014

PD: power of discrimination, A_e: effective number of alleles.

Supplementary Table S5. Pairs or groups of microhaplotypes that are calculated to be in linkage disequilibrium.

locus name	distance to next (bp)	A_e
**mh03KK-007**	204,451	2.96
mh03KK-008		2.16
mh04KK-028	35,976	1.75
mh04KK-015	9,748	1.97
**mh04KK-016**	7,381	2.02
mh04KK-017		2.01
**mh05KK-022**	9,534	2.60
mh05KK-078	194	1.36
mh05KK-079		1.99
mh06KK-080	857,264	1.03
**mh06KK-026**		1.21
mh10KK-083	296,449	1.17
mh10KK-084	9,031	1.00
mh10KK-085	2,252	2.00
**mh10KK-086**		2.21
**mh10KK-101**	4,305	2.60
mh10KK-088		1.11
**mh11KK-039**	53,100	2.28
mh11KK-040	17,764	1.39
mh11KK-089	1,164	2.21
mh11KK-041	12,536	2.25
mh11KK-090		1.84
mh12KK-093	45,731	1.50
**mh12KK-045**		1.57
**mh17KK-052**	2,223,325	2.92
mh17KK-053		2.47
mh22KK-303	878	2.00
**mh22KK-060**		2.97
